# Dynamically Cyclic Fe^2+^/Fe^3+^ Active Sites as Electron and Proton-Feeding Centers Boosting CO_2_ Photoreduction Powered by Benzyl Alcohol Oxidation

**DOI:** 10.34133/research.0567

**Published:** 2024-01-10

**Authors:** Ben Lei, Gaofeng Zhou, Zhongyou Gong, Chao Liu, Ying Zhou, Vitaliy P. Guro, Yanjuan Sun, Jianping Sheng, Fan Dong

**Affiliations:** ^1^ School of Resources and Environment, Institute of Fundamental and Frontier Sciences, University of Electronic Science and Technology of China, Chengdu 611731, China.; ^2^ Chengdu Zhihe Environmental Technology Co. Ltd., Chengdu 610207, China.; ^3^ School of Material Science and Engineering, Chongqing Jiaotong University, Chongqing 400074, China.; ^4^ School of New Energy and Materials, Southwest Petroleum University, Chengdu 610500, China.; ^5^ Institute of General and Inorganic Chemistry, Academy of Sciences of the Republic of Uzbekistan, Tashkent 100047, Uzbekistan.

## Abstract

Solar-driven CO_2_ photoreduction holds promise for sustainable fuel and chemical productions, but the complex proton-coupled multi-electron transfer processes and sluggish oxidation half-reaction kinetics substantially hinder its efficiency. Here, we devised a rational catalyst design to address these challenges by fabricating ferrocene carboxylic acid-functionalized Cs_3_Sb_2_Br_9_ nanocrystals (CSB-Fc NCs), which facilitate simultaneous benzyl alcohol oxidation and CO_2_ reduction reactions under visible-light irradiation. The synchronized proton-coupled electron transfer processes between the reduction and oxidation half-reactions on CSB-Fc NCs resulted in a 5-fold increase in the CO_2_ reduction rate (45.56 μmol g^−1^ h^−1^, 97.9% CO selectivity) and a 5.8-fold enhancement in benzyl alcohol conversion (97.7% selectivity for benzaldehyde) compared to the CSB. In situ Raman and ultraviolet-visible diffuse reflectance spectra revealed that the dynamic Fe^2+^/Fe^3+^ redox loop within the Fc unit serves as the actual active site, facilitating the activation of substrate molecules. More importantly, in situ attenuated total reflection Fourier transform infrared spectroscopy and gas chromatography–mass spectrometry spectroscopy, with isotope labeling of Deuteron-benzyl alcohol and ^13^CO_2_, confirmed that proton transfer from the hydroxyl group generates reactive protons at the Fe^2+^/Fe^3+^ site, enabling efficient CO_2_ photoreduction through subsequent protonation steps. This work offers a cost-effective and efficient approach for synergetic CO_2_ photoreduction driven by organic synthesis, advancing solar energy utilization.

## Introduction

Solar-driven CO_2_ photoreduction into usable fuels and value-added chemicals, employing H_2_O as the green proton and electron donor [1–4], is a vital strategy for storing solar energy within chemical bonds sustainably to combat the greenhouse effect and address the energy crisis [5–8]. However, the current state of the art in CO_2_ photoreduction is critically restricted by the sluggish H_2_O oxidation half-reaction [9–12], resulting in high reaction overpotentials and concomitant production of less valuable O_2_ production [13–15]. This O_2_ byproduct competes for electrons, further impeding the desired CO_2_ reduction process. While the incorporation of hole scavengers like triethanolamine can alleviate these challenges, they introduce additional reagent costs and inefficient utilization of the oxidizing power [16,17]. Therefore, exploring thermodynamically favorable oxidation half-reactions, such as organic synthesis, is paramount for enhancing overall photoredox efficiency [18–20].

The synergetic photocatalytic redox approach, which integrates CO_2_ reduction with organic synthesis [21–24], has attracted considerable attention due to its potential to improve overall photocatalytic efficiency. Achieving success with this approach requires precise control over several key factors, including charge separation [25,26], selective deprotonation of organic molecules, and efficient proton-coupled multi-electron transfer during CO_2_ reduction [27–30]. These prerequisites highlight the need for advanced photocatalyst designs. Recent research has focused on the development of composite catalysts that incorporate both oxidative and reductive sites to optimize charge utilization and product selectivity. This optimization is achieved by regulating carrier kinetics, lowering activation barriers, and controlling intermediate species migration [22,24]. However, the presence of an interfacial barrier between these spatially separated active sites can hinder efficient electron and proton delivery, particularly in proton delivery, due to the complexity of multiple electron and proton transfers in synergetic photoredox reactions [27,31]. These challenges constrain further enhancements in overall photocatalytic efficiency and product selectivity. Thus, it is crucial to minimize steric hindrance and improve electron–proton transport kinetics by creating isolated active sites with simultaneous redox capacities.

Recently, ferrocene (Fc)-based complexes have garnered extensive attention in the fields of photovoltaic and catalysis, owing to their exceptional properties such as extended light absorption range and rapid charge transfer capabilities [32,33]. These Fc-based complexes can serve as efficient and stable electron donors, capitalizing on their electron-donating ability to efficiently transfer electron density from their delocalized π-electrons in the cyclopentadienyl rings to the *d*-orbitals of Fe atoms [34]. Furthermore, Fc serves as a well-established redox mediator, characterized by reversible redox properties exemplified by the Fc/Fc^+^ couple, which enhances electronic interaction and transfer at the catalytic interface [33,35]. Despite these advantageous features, the majority of studies have predominantly focused on the influence of Fc on charge transfer, often overlooking the potential redox capability of the reversible Fe^2+^/Fe^3+^ site associated with the Fc/Fc^+^ redox couple. Additionally, the mechanisms of intermolecular proton migration, which are critical to understanding deprotonation and protonation processes in both organic synthesis and CO_2_ reduction half-reactions, have not been thoroughly investigated. The dynamic equilibrium and migration of H^+^ protons during photoredox reactions remain poorly explored, further complicating the mechanistic insights required for efficient catalytic design. Therefore, there is an urgent need, albeit a substantial challenge, to develop isolated and reversible Fe^2+^/Fe^3+^ redox sites capable of simultaneously enhancing the oxidation and reduction half-reaction using Fc as a building block. This innovative approach would involve improving electron–proton transport kinetics, revealing the electron–proton migration mechanism, and ultimately increasing the activity and selectivity of CO_2_ reduction coupled with organic synthesis.

**Fig. 1.**
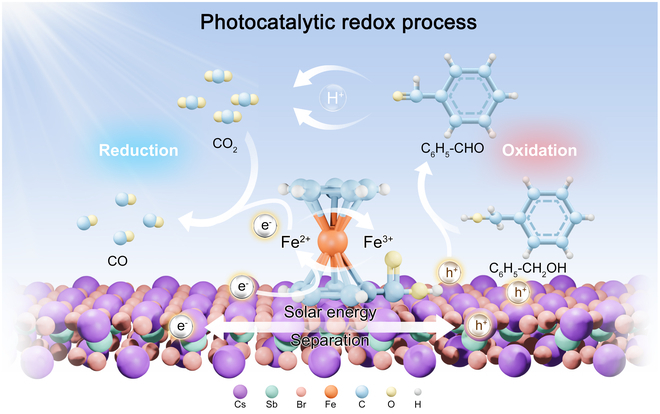
Schematic diagram of the proton-coupled electron transfer mechanism driven by Fe^2+^/Fe^3+^ loop site on CSB-Fc NCs during the photocatalytic redox process.

Here, we propose a rational catalyst construction strategy based on perovskite nanocrystal (NC) ligand engineering. Taking Cs_3_Sb_2_Br_9_ (CSB) NCs as prototype catalysts, Fc-functionalized CSB NCs enable efficient and highly selective synergistic catalysis, simultaneously facilitating CO_2_ reduction and organic molecule oxidation (Fig. 1). Importantly, in situ Raman and ultraviolet-visible diffuse reflectance spectra (UV-vis DRS) spectra confirm the variability of the Fe^2+^/Fe^3+^ loop within Fc, serving as a dynamically active site that promotes the activation and conversion of substrate CO_2_ and benzyl alcohol molecules. As expected, the photoreduction of CO_2_ integrated with benzyl alcohol oxidation over CSB-Fc NCs proceeds efficiently, yielding approximately 45.56 μmol g^−1^ h^−1^ of CO and high selectivity for both produced benzaldehyde (97.7%) and CO (97.9%). In addition, the application of isotope-labeled in situ attenuated total reflection Fourier transform infrared spectroscopy (ATR FT-IR) and gas chromatography–mass spectrometry (GCMS) spectra utilizing both Deuteron-benzyl alcohol and ^13^CO_2_ provides compelling evidence that hydrogen ions dissociated from the hydroxyl groups of the benzyl alcohol are preferentially activated on the electron-rich Fc sites. These reactive protons directly participate in the following protonation of CO_2_ through the proton-coupled electron transfer process. These dynamically cyclic Fe^2+^/Fe^3+^ active sites act as co-feeding centers for electrons and protons, making the simultaneous oxidation and reduction reactions effectively occur and enhancing the overall catalytic efficiency by reducing the energy barriers. This work opens new avenues for the efficient conversion of solar energy into fuels and chemicals, showcasing exceptional performance and cost-effectiveness.

## Results

### The structure feature of catalysts

**Fig. 2.**
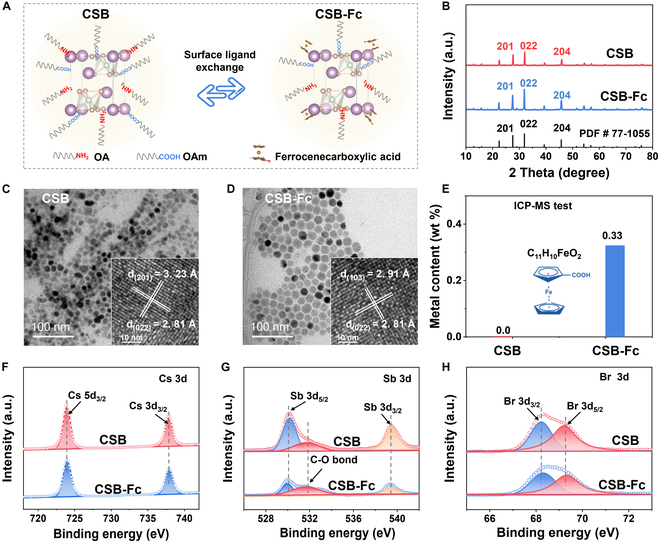
Structure feature of as-prepared samples. (A) Schematic diagram of surface ligand exchange. (B) XRD spectra. (C and D) TEM image and HRTEM image of CSB NCs and CSB-Fc NCs. (E) ICP-MS test. (F to H) XPS spectra of Cs 3d, Sb 3d, and Br 3d.

We synthesized the CSB NCs capped with oleylamine and oleic acid using a hot injection method, followed by ligand exchange with Fc ligand to functionalize the surface. The strong interaction between the short-chain Fc ligand and the NC surface facilitates the replacement of the long-chain oleylamine and oleic acid ligands (Fig. S1). A schematic diagram of the surface ligand exchange process is shown in Fig. 2A. The crystal structures of both CSB and CSB-Fc NCs consist of typical octahedral (Sb_2_Br_9_)^3−^ clusters, with interlayer gaps filled by Cs^+^ ions. As depicted in Fig. 2B, the x-ray diffraction (XRD) spectra display that all the diffraction peaks of CSB and CSB-Fc NCs can be readily indexed to standard trigonal CSB NCs (PDF# 77-1055) [36], indicating that the crystal structure remains intact after ligand exchange. Transmission electron microscopy (TEM) and high-resolution TEM (HRTEM) reveal a uniform size distribution for the monodispersed CSB NCs, with an average diameter of 14.91 ± 0.6 nm (Fig. 2C and Fig. S2A). The 2 sets of lattice spacings are 2.81 and 3.23 Å, respectively, corresponding to the (022) and (201) planes of CSB NCs, which are well in agreement with the identified structure of XRD standard cards. After ligand exchange, the average particle size of CSB-Fc NCs slightly increased to 17.87 ± 0.5 nm, as shown in Fig. 2D and Fig. S2B. It can be deduced that the long-chain oleylamine and oleic acid may substantially contribute to the maintenance of structural integrity compared to the small-chain ligands that NCs undergo aggregation. The exposed crystal plane of CSB-Fc NCs is similar to those of CSB NCs and tends to be exposed on the (022) plane. Interestingly, inductively coupled plasma mass spectrometry (ICP-MS) indicates the successful functionalization of CSB with Fc, showing the Fe content of 0.33% in CSB-Fc NCs, while no Fe element was detected in CSB NCs (Fig. 2E). As a result, it can be inferred that short-chained Fc-decorated CSB NCs have been successfully prepared using a straightforward method.

The x-ray photoelectron spectroscopy (XPS) was employed to investigate the chemical states of as-prepared samples. The characteristic peaks of Cs 3d are observed at 723.9 and 737.9 eV for CSB and CSB-Fc NCs, respectively, corresponding to Cs 3d_5/2_ and Cs 3d_3/2_ (Fig. 2F) [37]. Additionally, 2 peaks at 530.14 and 539.7 eV, respectively, correspond to Sb 3d_3/2_ and Sb 3d_5/2_ (Fig. 2G). Notably, the O 1s peak appears at a lower binding energy in the Sb 3d spectrum, attributed to the C–O bonding of the surface ligand. Furthermore, the Br 3d peaks exhibit consistent binding energy peaks at 68.3 and 69.5 eV, assigned to Br 3d_3/2_ and Br 3d_5/2_, respectively (Fig. 2H) [37]. The stability of these peaks confirms minimal bonding interactions between Cs and Br ions, whether on the surface or in the crystal lattice. Moreover, the XPS survey spectra indicate the elemental composition of these samples, comprising Cs, Sb, C, O, and Br elements (Fig. S3), further corroborating the structural integrity and successful surface modification of the NCs.

### Active sites and carrier dynamics

**Fig. 3.**
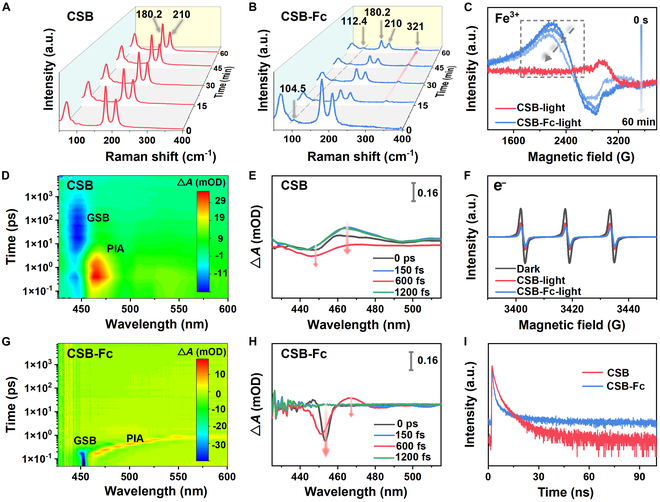
Carrier dynamics and optical properties of CSB and CSB-Fc NCs. (A and B) In situ Raman spectra. (C) EPR spectra. (D and E) Femtosecond TA plot. (F) EPR spectra of e^–^. (G and H) TA spectra at the indicated delay time from 0 to 1,200 fs. (I) Time-resolved photoluminescence.

**Fig. 4.**
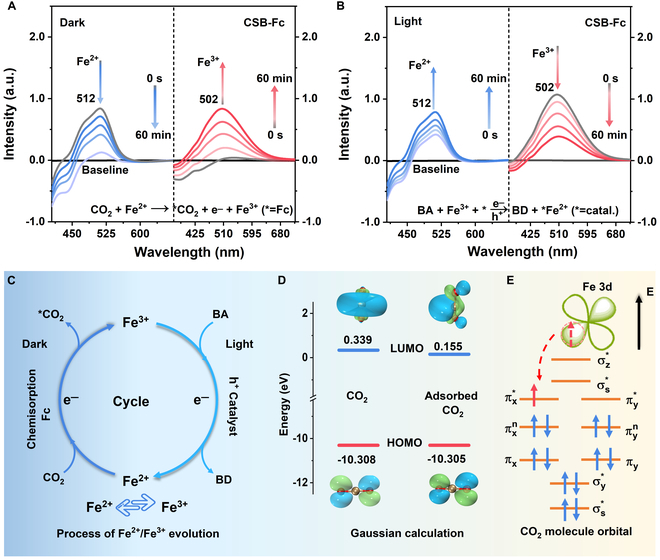
(A and B) UV-vis DRS spectra of CSB-Fc under dark and light irradiation for adsorption process and photocatalytic reaction. (C) Conversion pathway of Fe^2+^/Fe^3+^. (D) LUMO and HOMO energy of CO_2_ before and after adsorption. (E) CO_2_ molecule orbital.

We conducted in situ Raman spectra on as-prepared samples using a custom-built setup to identify the reactive active site for catalytic activity. For CSB NCs, 2 distinct bands at 180.2 and 210 cm^−1^ were detected, corresponding to the vibrations of Sb-Br_2_ and Sb-Br_6_ bonds, respectively (Fig. 3A). These bonds remained unaltered upon injection of CO_2_ and benzyl alcohol under both dark and light irradiation conditions. In contrast, CSB-Fc NCs exhibit a new Raman band at 321.0 cm^−1^, characteristic of A_1_g ring metal stretch [38,39], suggesting that Fc-decorated NCs facilitate the chemisorption and activation of substrate molecules (Fig. 3B). Intriguingly, the Fe–Cp bond at 104.5 cm^−1^ was shifted to 112.4 cm^−1^ in Fc species, and its strength progressively increased over time during light irradiation. Furthermore, as depicted in Fig. 3C, the electron paramagnetic resonance (EPR) spectrum of CSB NCs exhibits no apparent signals, whereas CSB-Fc NCs display characteristic signals of Fe^3+^ under light irradiation. The intensity of these signals gradually decreased over time, attributed to the reduction of Fe^3+^ to diamagnetic Fe^2+^ through the trapping of photoelectrons from CSB NCs. The mechanism of interconversion between Fe^3+^ and Fe^2+^ species was further revealed by subsequent characterizations of UV-vis DRS spectra and Gaussian calculations (Fig. 4).

Femtosecond transient absorption (TA) spectroscopy was employed using CSB and CSB-Fc NCs as a model system to investigate the dynamics of carriers and photophysical properties following ligand exchange. The experiments were conducted with 400-nm excitation (3.1 eV) and a pump power of 25 μW. As shown in Fig. 3D, the pseudocolor TA plot of CSB NCs shows a ground-state bleach (GSB) at 450 nm, which can be attributed to state-filling-induced bleach [40]. Additionally, a photo-induced absorption (PIA) band, detected in the red-orange region (465 to 485 nm), is possibly arising from hot carriers induced by absorption in excited states. In contrast, CSB-Fc NCs exhibit a broad PIA signal extending across the probe region (red-orange region, 465 to 600 nm), corresponding to a long-lived positive PIA at higher energies (Fig. 3G). The negative bleaching signal at 450 nm is attributed to the sub-bandgap state absorption [41,42]. These observations suggest that Fc-decorated CSB NCs introduce new band states within the original energy bands, leading to broadened photoluminescence emission at longer wavelengths. As seen in Fig. 3E and H, both PIA signals of CSB and CSB-Fc NCs are observed to be red-shifted, remarkably extending the light absorption ranges [43]. The negative amplitude of the GSB signal for CSB-Fc NCs is 2-fold higher than that of CSB NCs at 450 nm. This increase in bleach amplitudes indicates enhanced carrier generation upon photoexcitation in CSB-Fc NCs. Surprisingly, EPR spectra for CSB, Fc, and CSB-Fc NCs displayed similar signals of captured photoelectrons in the dark (Fig. 3F and Fig. S4). Under light irradiation, the signal of captured photoelectrons for Fc species disappeared, suggesting the Fc species act as a special electron donor–acceptor conjugate structure (Fig. S4), which can achieve remarkably prolonged times of charge separation. Following surface ligand exchange, the signal of captured photoelectrons for CSB-Fc NCs is lower than that of CSB NCs, suggesting more efficient charge separation. As can be seen from time-resolved photoluminescence (PL) in Fig. 3I, the decay of photogenerated charges in CSB-Fc NCs is distinctly lower than that in CSB NCs. This decay process can be accurately fitted by a double-exponential function comprising a short-lived component (τ_1_ = 4.81 ns) and a long-lived component (τ_2_ = 0.43 ns). The average photoluminescence decay of CSB-Fc NCs was approximately 0.46 ns, notably lower than that of CSB NCs (1.407 ns) (Table S1). This reduction in lifetime reflects strong electronic coupling between Fc and CSB NCs, facilitating rapid charge transfer after ligand exchange. Moreover, steady-state PL intensity measurements reveal an obvious reduction in the emission of CSB-Fc NCs compared to CSB NCs, indicative of more efficient electron-hole pair separation (Fig. S5). Furthermore, UV-vis diffuse reflectance spectroscopy (DRS) revealed a substantial alteration in the light absorption properties of CSB NCs after Fc incorporation. A slight red shift in the absorption edge of CSB-Fc NCs, coupled with the extension of its light absorption range into the infrared spectrum, indicates an improvement in light absorption capability (Fig. S6A). The corresponding bandgap energy reduced from 2.42 eV in CSB NCs to 2.40 eV in CSB-Fc NCs (Fig. S6B).

Moreover, density functional theory (DFT) calculations were employed to simulate the density of state (DOS) and partial density of states (PDOS) for CSB and CSB-Fc NCs, providing insights into the impact of Fc on the energy band structure (Fig. S7A to C). The bandgap of CSB-Fc NCs exhibits a slight shift toward the conduction band compared to CSB NCs. Subsequently, PDOS results further confirmed that the hybridization of Sb 3d and Fe 3d orbitals predominantly contributes to the valence band in CSB-Fc NCs, suggesting that Fe plays a critical role in the observed electronic modulation. Based on these insights, we propose band structure for CSB-Fc NCs (Fig. S7D), where Fc-functionalized CSB NCs can generate a sub-bandgap state (as evidenced by femtosecond TA spectroscopy) [41,42]. The efficient transfer of photogenerated charges into the sub-bandgap, with electrons occupying the Fe 3d orbitals on the surface of Fc, markedly reduces the recombination of electron-hole pairs. This enhanced charge separation was corroborated by photoelectrochemical measurements, which demonstrate that the photocurrent intensity of CSB-Fc NCs is substantially higher than that of CSB NCs (Fig. S8). Electrochemical impedance spectroscopy, conducted at a bias voltage of 0 V across a frequency range of 0.1 to 10^5^ Hz, revealed that CSB-Fc NCs exhibited smaller semicircles than CSB NCs, signifying lower charge-transfer resistance and superior carrier transport properties (Fig. S9). Collectively, these findings suggest that Fc-decorated CSB NCs generate more photo-induced charge carriers, thereby enhancing their participation in subsequent redox reactions. In addition, to further investigate the interactions between adsorbed species and catalysts, the activation behaviors of CO_2_ on the CSB-Fc NCs surface were explored via DFT calculation. A stable and optimal module structure is determined by DFT calculation after the exchange of the Fc ligand (Figs. S10 and S11). The adsorption energy of *CO_2_ and C_6_H_5_-CH_2_OH on CSB-Fc NCs atomic layers is −23.38 and −99.858 eV, respectively, which was lower than that of those on CSB NCs (−23.36 and −99.855 eV) (Figs. S12 and S13). These DFT results imply a robust mutual interaction between the adsorbate and the CSB-Fc surface, which promotes more effective activation of CO₂ and benzyl alcohol, making the catalytic process more favorable.

To investigate the interconversion between Fe^3+^ and Fe^2+^ species, UV-vis DRS was employed in conjunction with potassium thiocyanate (KSCN) and o-phenanthroline colorimetric methods [44–46]. Under dark conditions, the strongest absorbance signal at 512 nm is observed [45], corresponding to Fe^2+^ ions present in Fc-functionalized NCs after surface ligand exchange (Fig. 4A). Upon injection of CO_2_ gas, the intensity of Fe^2+^ absorbance signal weakened, while the signal at approximately 501 nm (assigned to Fe^3+^ species) increased, suggesting the conversion of Fe^2+^ to Fe^3+^ during the reaction with CO_2_. Under light irradiation, we observed opposing variations in the absorption intensity of Fe^3+^ and Fe^2+^ (Fig. 4B). This indicates that Fe^3+^ actively captures the photoexcited electrons from the catalyst, leading to their reduction to Fe^2+^. The addition of benzyl alcohol, acting as a sacrificial agent, is activated by cavitation to enhance this redox cycle, as shown schematically in Fig. 4C.

For further exploration of the interconversion mechanism between Fe^3+^ and Fe^2+^, we conducted calculations on the lowest occupied hybrid orbital (LUMO) and highest occupied molecular orbital (HOMO) of CO_2_ by Gaussian calculation, along with the molecular hybrid orbital of CO_2_ (Fig. 4D). Initially, HOMO and LUMO energy of CO_2_ are −10.308 and 0.339 eV, respectively. The LUMO energy of adsorbed CO_2_ was reduced to about 0.155 eV, indicating an enhanced propensity for CO₂ activation at the active sites. According to the molecular orbital theory [47], it is deduced that LUMO of CO_2_ could be involved in the CO_2_ chemisorption process. The conversion of Fe^2+^ to Fe^3+^ occurred easily, which is attributed to the presence of a solitary electron in the 3d nonbonding orbital of Fe^2+^ in Fc (Fig. 4E). This lone electron occupies the lowest energy level of the π-bond of the CO_2_ molecule, facilitating the chemisorption reaction of Fc with CO_2_ and enabling oxidation to Fe^3+^. Subsequently, the catalyst is photoexcited to generate electron-hole pairs, with benzyl alcohol acting as a sacrificial agent to deplete the holes. Fe^3+^ then captures the photogenerated electrons, occupying all the unoccupied orbitals of LUMO in the Fc species (Fig. S14). Based on the above results, it can be deduced that the variable valence forms of Fe^2+^/Fe^3+^ in Fc species serve as actual active sites, driving the activation of adsorbed reactant molecules through efficient electron and proton transfer processes during the photocatalytic reaction.

### Catalytic activity and reactive mechanism

**Fig. 5.**
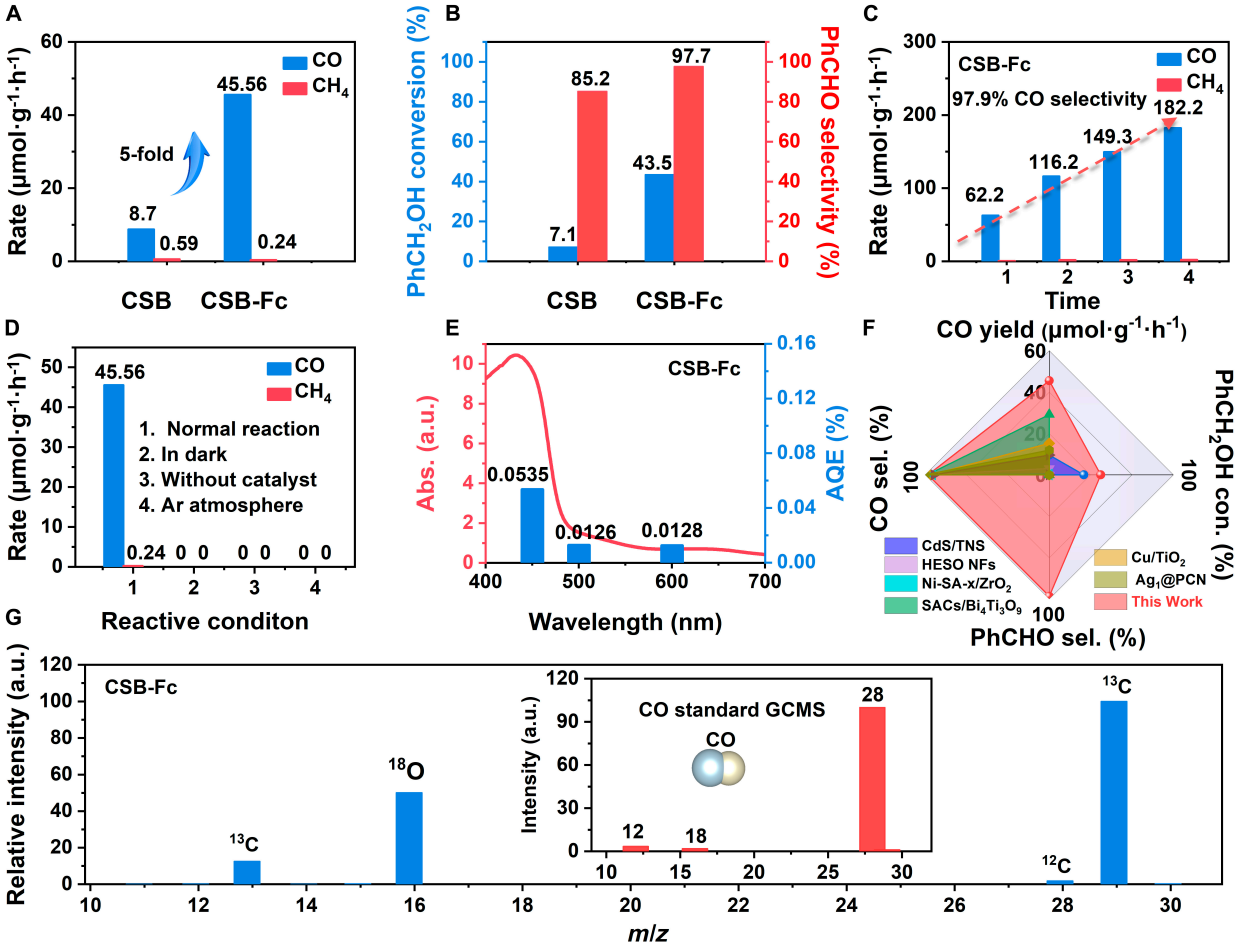
(A) Photoredox activity: photoreduced CO_2_ of CSB and CSB-Fc NCs. (B) Conversion of benzyl alcohol and selectivity of benzaldehyde. (C) Gas amount of CSB-Fc. (D) Different reactive conditions. (E) AQE and UV-vis DRS spectra. (F) Compared with lately developed catalysts and their performance, all references can be found in Table S5. (G) GCMS spectra of ^13^CO_2_ isotope experiments.

The photocatalytic efficiency for CO_2_ reduction coupled with selective photooxidation was systematically evaluated under visible light irradiation for the as-prepared samples. As depicted in Fig. 5A, the CSB NCs achieved a modest CO yield of 8.7 μmol g^−1^ h^−1^ with minimal CH_4_ production, and the benzyl alcohol conversion reached only 7.1% after 4 h. In contrast, CSB-Fc NCs exhibited remarkable performance, yielding 45.56 μmol g^−1^ h^−1^ of CO, approximately 5-fold higher than that of CSB NCs. Simultaneously, the conversion of benzyl alcohol over CSB-Fc NCs reached 43.5%, predominantly yielding the target product (benzaldehyde), with only trace amounts of benzoic acid. The selectivity for benzaldehyde was 97.7%, nearly 5.8 times higher than that of the CSB NCs (Fig. 5B and Tables S2 and S3). As shown in Fig. 5C, the CSB-Fc NCs demonstrated a linear increase in CO production, achieving a remarkably high selectivity of 97.9%. Control experiments performed in the dark or without a photocatalyst yielded no products (Fig. 5D). Similarly, replacing CO_2_ with Ar gas only led to undetectable levels of CO and CH_4_ production with CSB-Fc NCs. Interestingly, the apparent quantum efficiency (AQE) is correlated with the absorption spectrum. The AQE of CSB-Fc NCs is 0.535% in the CO_2_ photoreduction at 450 nm (Table S4 and Fig. 5E). Compared with recently developed photocatalysts, CSB-Fc NCs demonstrate superior performance in both activity and selectivity (Table S5 and Fig. 5F) [18,23,48–50]. The photocatalytic performance of this catalyst still maintained relative stability in a 3-cycle test (Figs. S15 and S16). Furthermore, to confirm the carbon source of the CO product, isotopic labeling experiments using ^13^CO_2_ were conducted (Fig. 5G), where the strong relative intensity of ^13^C [mass/charge ratio (*m*/*z*) = 29] confirmed that the CO originated precisely from the photocatalytic reduction of CO_2_ rather than from the catalyst.

### Reaction pathways and mechanism

The dynamic evolution of reactants and intermediates on the sample surfaces was probed over time using in situ ATR FT-IR, allowing for a detailed exploration of the photoredox reaction mechanism. The adsorption processes induced by the injection of CO_2_ gas and benzyl alcohol in the dark for both CSB and CSB-Fc NCs are illustrated in Fig. S17. The emergence of IR bands at 3,436 and 1,334 cm^−1^, corresponding to hydroxyl group of benzyl alcohol and b-CO_3_^2−^, respectively [51], confirms the accumulation of these adsorbed products on the surface of CSB NCs. In contrast, the CSB-Fc NCs exhibited additional peaks, including m-CO_3_^2−^ at 2,933 cm^−1^ [52], indicating a visibly enhanced adsorption affinity of these substrates on the catalyst surface.

**Fig. 6.**
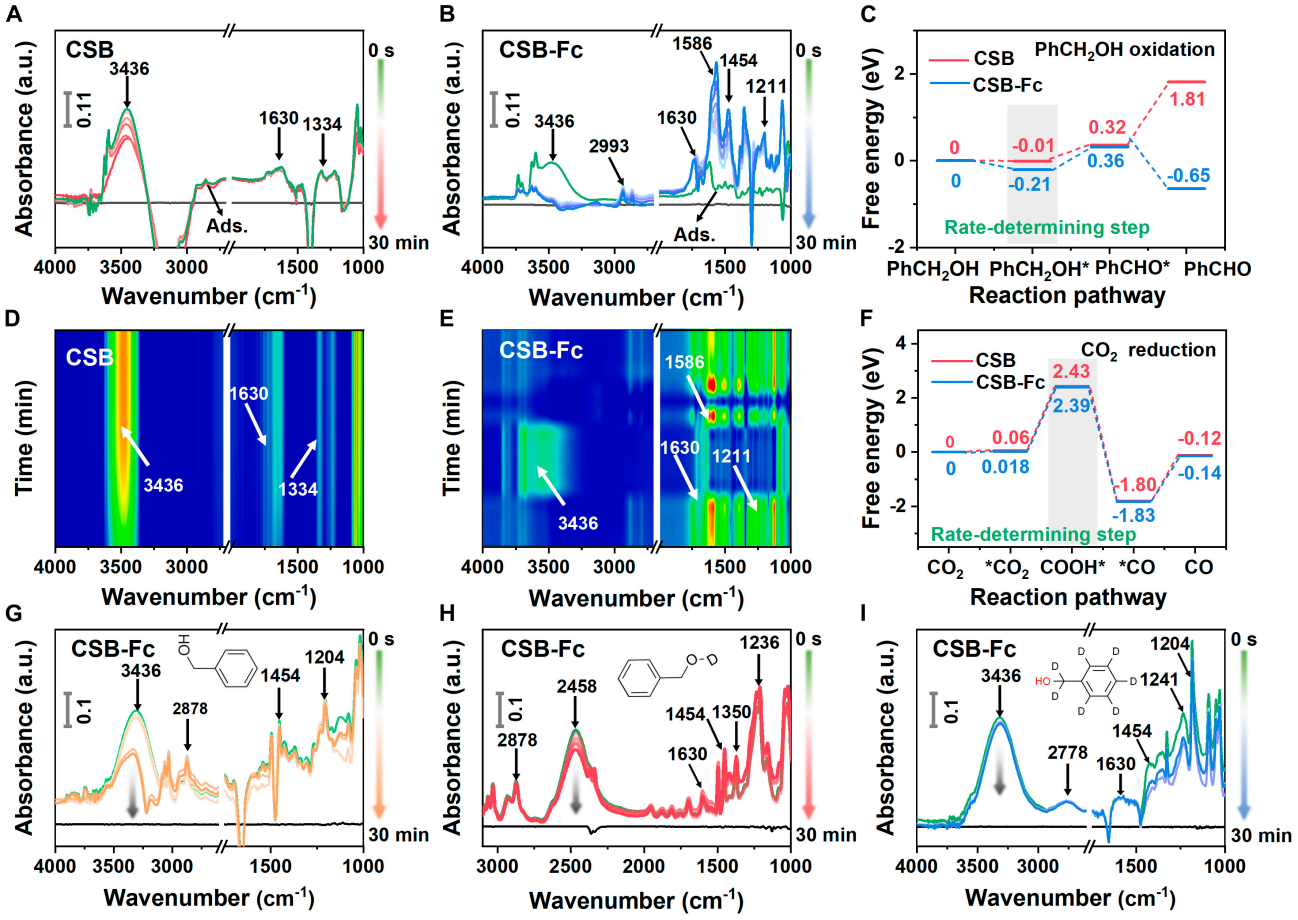
In situ ATR FT-IR for the reactive process under light irradiation. (A) CSB NCs. (B) CSB-Fc NCs. (D and E) Contour map for IR data. (C and F) Gibbs free energy (∆*G*) of C_6_H_5_-CH_2_OH oxidation and CO_2_ reduction over CSB and CSB-Fc NCs. (G to I) Isotope labeling experiments of benzyl alcohol, benzyl alcohol-OD, and benzyl-D7 alcohol.

After reaching adsorption equilibrium within 30 min, time-dependent in situ ATR FT-IR spectra were recorded dynamically under light irradiation for both CSB and CSB-Fc NCs. The infrared contour maps indicate a slight reduction in the IR band corresponding to benzyl alcohol, while other adsorbed substrates, particularly bicarbonate (b-CO_3_^2−^), remained stable on CSB NCs (Fig. 6A and D). This observation suggests limited oxidation of the adsorbed reactants. In contrast, for CSB-Fc NCs, the band of benzyl alcohol (at 3,436 cm^−1^) was obviously reduced and depleted, accompanied by the emergence of a new band at 1,630 cm^−1^ corresponding to benzaldehyde [51,53], indicating effective oxidation of benzyl alcohol during illumination (Fig. 6B and E). Additionally, new IR bands at 1,211 and 1,454 cm^−1^ (attributed to ·COOH) were observed, serving as important intermediates in the photoreduction of CO_2_ to CO [52,54,55]. The IR peak at 1,586 cm^−1^ (attributed to b-CO_3_^2−^) was also observed, resulting from the asymmetric O–C–O stretching of the bidentate and monodentate carbonate groups. These results suggest that CSB-Fc NCs can oxidize benzyl alcohol to benzaldehyde while simultaneously facilitating the reduction of CO_2_ to the key intermediate (*COOH).

The isotopic labeling experiment was further carried out by in situ ATR FT-IR spectra to confirm the source and transfer process of hydrogen protons. Figure 5G to I illustrates the in situ ATR FT-IR spectra involving benzyl alcohol, benzyl alcohol-OD, and benzyl-D7 alcohol. The focus was on vibrations between the O and H(D) atoms of the benzene ring. The characteristic peak of the out-of-plane wobble vibration of the hydroxyl group in benzyl alcohol is located at 3,436 cm^−1^, while in benzyl alcohol-OD, this peak shifted to 2,458 cm^−1^, both exhibiting similar weakening trends. Concurrently, both characteristic bonds of 1,454 and 1,204 cm^−1^ (attributed to ·COOH) shifted to 1,350 cm^−1^ (assigned to COOD vibrations) [52,54] (Fig. 6G and H). Additionally, both peaks for *CO_2_ (1,236 cm^−1^) [56] and the corresponding aldehyde (1,630 cm^−1^) are detected. Interestingly, the stretching vibration of C–H in benzyl alcohol is detected at 2,878 cm^−1^, while the IR peak of C–D in benzyl-D7 alcohol shifted to 2,778 cm^−1^ (Fig. 6G and I). These IR peaks of *CO_2_ (1,241 cm^−1^) and COOH* (1,204 and 1,454 cm^−1^) were accumulated on the catalyst surface, whereas no characteristic COOD peaks were observed. Thus, these results directly confirm that the hydroxyl group of the benzyl alcohol can serve as a proton source, driving the photocatalytic high-selectivity conversion of CO_2_ to CO.

Following the preceding discussion, the Gibbs free energy (∆*G*) calculations were performed using DFT to elucidate the potential photoredox reaction pathway. For the oxidation of C_6_H_5_-CH_2_OH on CSB NCs, the ∆*G* value (−0.01 eV) of C_6_H_5_-CH_2_OH* indicates an exothermic and spontaneous process. Interestingly, the ∆*G* value for C_6_H_5_-CH_2_OH* over CSB-Fc NCs (−0.21 eV) is lower, suggesting that the conversion of C_6_H_5_-CH_2_OH to C_6_H_5_-CH_2_OH* is the rate-determining step in the photooxidation reaction. The ∆*G* values for C_6_H_5_-CHO* on both CSB and CSB-Fc NCs are 0.36 and 0.32 eV, respectively, indicating that the dissociation of hydrogen from benzyl alcohol to form benzaldehyde is an endothermic reaction requiring a small energy barrier to be overcome (Fig. 6C). The subsequent ∆*G* value for C_6_H_5_-CHO is −0.65 eV on CSB-Fc NCs, much lower than that on CSB NCs, implying that C_6_H_5_-CHO* is very easily desorbed to form free C_6_H_5_-CHO molecules. Thus, the conversion of C_6_H_5_-CH_2_OH to C_6_H_5_-CHO is the selectivity-determining step in the photooxidation reaction.

Meanwhile, for the CO_2_ photoreduction (Fig. 6F), the ∆*G* value of *CO_2_ on CSB-Fc NCs (0.018 eV) is lower than that on CSB NCs (0.06 eV), indicating that activation of CO_2_ to *CO_2_ on CSB-Fc faces only a small energy barrier. The formation of *COOH on both CSB and CSB-Fc NCs is an endothermic process, with CSB-Fc NCs exhibiting a lower energy barrier (∆*G* value = 2.39 eV) compared to CSB NCs (∆*G* value = 2.43 eV). It is inferred that *COOH reacts with protons and electrons to produce *CO molecules, which is the rate-determining step. The *CO group can then spontaneously desorb from the CSB-Fc NCs surface to generate free CO molecules. As a result, CSB-Fc NCs can leverage substantial modulation effects of surface ligand exchange to facilitate the directed transformations of adsorbed molecules, optimizing the rate-controlling step and enhancing the overall efficiency of the photoredox reaction.

## Discussion

In summary, the simultaneous execution of CO_2_ photoreduction and oxidative organic photosynthesis was successfully realized through the strategic design of catalysts modified with Fc surface ligands. The integration of the Fc ligand effectively minimized the spatial delivery distance of photogenerated electron transfer, thus effectively enhancing carrier transport efficiency. The Fe^3+^/Fe^2+^ redox couple within Fc ligands served as a pivotal active center, facilitating the activation of substrate molecules. As a result, Fc-functionalized CSB NCs demonstrated exceptional photocatalytic activity, achieving a CO production rate of approximately 45.56 μmol g^−1^ h^−1^, with a selectivity of exceeding 97.7% for both CO and benzaldehyde. Notably, isotope-labeled in situ ATR FT-IR spectra, GCMS, and DFT calculations revealed that hydrogen protons generated from the oxidative half-reaction play a crucial role in driving the protonation process during CO₂ reduction. This innovative approach offers profound insights into the design and industrial application of synergistic photoredox catalytic systems, laying a solid foundation for solar-driven energy conversion and environmental catalysis.

## Materials and Methods

### Reagents

All the chemicals were analytically pure without further processing.

### Catalyst preparation

The CSB NCs were synthesized using the modified hot injection method [36]. First, a mixture of 2.0 mmol of Cs_2_CO_3_, 2.5 ml of oleic acid, and 20.0 ml of 1-octadecene was degassed under vacuum at 120 °C for 1 h and then heated at 150 °C under the N_2_ atmosphere to obtain the Cs-oleate precursor. Second, a mixture of 10.0 ml of 1-octadecene, 0.6 ml of oleylamine, 1.0 ml of oleic acid, and 0.1 mmol of SbBr_3_ was packed into a 3-neck flask and degassed for 1 h at 80 °C under vacuum. Following heating to 180 °C under an N_2_ atmosphere, 0.3 ml of the previously prepared Cs-oleate solution was rapidly injected under vigorous stirring. After the reaction time (60 s), the reaction mixture was cooled in an ice water bath. This solution was centrifuged at 7,800 rpm for 5 min to separate precipitates and supernatant. Finally, after the addition of 5.0 ml of hexane, the supernatant was centrifuged at 12,500 rpm for 5 min to obtain the CSB NCs.

#### Synthesis of CSB-Fc NCs

Surface ligand exchange was carried out in an air atmosphere. A specific amount of Fc-carboxylic acid (1 ml, 0.035 M) was dissolved in 2 ml of hexane containing a solution of hydrogen bromide. After full dissolution, 5 ml of the CSB NCs solution, as mentioned above, was added and stirred for 30 min. After the surface ligand exchange was completed, the mixed solution was centrifuged, washed, and dried in a vacuum oven at 60 °C. The obtained yellow powder samples were labeled as CSB-Fc NCs.

### Detection of Fe(II)/Fe(III) ions

The determination of Fe^2+^/Fe^3+^ was carried out by scanning the UV-vis spectrum (Shimadzu UV-2450) using the KSCN and o-phenanthroline colorimetric method [44–46]. In our work, the generation of Fe^2+^/Fe^3+^ plays a crucial role in the photocatalytic redox reaction. The complexation Fe^2+^/Fe^3+^ with o-phenanthroline colorimetric and KSCN was employed for color development, respectively [57]. The mechanism is that Fe^2+^ and o-phenanthroline ionic complexes form stable orange-red tri-ligand complexes at pH 2.5 to 9.0. Accordingly, the absorbance and characteristic absorption peaks were measured at 510 nm. Only divalent iron forms an orange complex with o-phenanthroline, while trivalent iron does not undergo a chromogenic reaction, thus making absorbance proportional to the concentration of Fe^2+^. For photocatalytic redox reactions, 1 ml of solution was mixed with 5 ml of acetic acid–sodium acetate buffer solution (1 mM), followed by the addition of 2 ml of 0.5% o-phenanthroline solution in a cuvette (10 ml). The resulting mixed solution was then analyzed using a UV-visible spectrophotometer. Similarly, the concentration of Fe^3+^ was detected using the same method but substituting KSCN (5 mg ml^−1^) as the developer. The Fe^3+^ concentrations in the catalytic reaction systems were determined following a procedure similar to the one described above.

### In situ ATR FT-IR investigation

In situ ATR FT-IR spectrometer is equipped with a narrowband HgCdTe detector and a diamond crystal ATR sampling plate (Harrick ConcentratIR 2). Fresh catalysts and reactant samples were dissolved in an acetonitrile solution, and 5 ml of the resulting suspension was dropped onto the diamond ATR crystal cell (Scheme S1). CO_2_ gas was then introduced into the reaction chamber, and the changes in reactants and intermediates over time during the reaction were monitored by ATR FT-IR spectroscopy. Isotope labeling experiments of benzyl alcohol-OD and benzyl-D7 alcohol were determined following a similar procedure as described above.

## Data Availability

All data needed for this study are available in the article and its Supplementary Materials.

## Supplementary Materials

Supplementary 1 Materials and Methods Scheme S1 Figs. S1 to S21 Tables S1 to S6
