# Supplementary material for: Dynamically Cyclic Fe2+/Fe3+ Active Sites as Electron and Proton-Feeding Centers Boosting CO2 Photoreduction Powered by Benzyl Alcohol Oxidation
Source: Research (Wash D C). 2024 Jan 10;8:0567. doi: 10.34133/research.0567 (PMC11717996; doi:10.34133/research.0567)
Supplement: Supplementary 1 — Materials and Methods Scheme S1 Figs. S1 to S21 Tables S1 to S6 [file research.0567.f1.docx]

Supporting Information for

Dynamically Cyclic Fe^2+^/Fe^3+^ Active Sites as Electron and Proton-Feeding Centers Boosting CO_2_ Photoreduction Powered by Benzyl Alcohol Oxidation

Ben Lei, ^1,3^ Gaofeng Zhou, ^1^ Zhongyou Gong, ^2^ Chao Liu, ^2^ Ying Zhou, ^4^ Vitaliy P. Guro,^5^ Yanjuan Sun,^1,^* Jianping Sheng,^1,^* and Fan Dong^1^

^1.^ Yangtze Delta Region Institute (Huzhou), University of Electronic Science and Technology of China, Huzhou 313001, China.

^2.^ Chengdu Zhihe Environmental Technology Co., LTD.; Chengdu 610207, China.

^3.^ School of Materials Science and Engineering, Chongqing Jiaotong University, Chongqing 400074, China.

^4.^ School of New Energy and Materials, Southwest Petroleum University, Chengdu 610500, China.

^5.^ Institute of General and Inorganic Chemistry, Academy of Sciences of the Republic of Uzbekistan, Tashkent 100047, Uzbekistan.

* Corresponding authors: Jianping Sheng: [jpshengchn@163.com](mailto:jpshengchn@163.com); Yanjuan Sun: sunyj@uestc.edu.cn

Table of CONTENT

[Materials and Methods 5](#_Toc183897286)

[Characterizations 5](#_Toc183897287)

[DFT calculations 6](#_Toc183897288)

[Evaluation of photo-redox activity 7](#_Toc183897289)

[In situ Raman tests 8](#_Toc183897290)

[Scheme S1. The designed reaction system for in situ ATR-FTIR signal recording. 9](#_Toc183897291)

[Fig. S1. The surface ligand length. 9](#_Toc183897292)

[Fig. S2. Analysis of the size distribution over CSB and CSB-Fc NCs 9](#_Toc183897293)

[Fig. S3. The survey spectra of as-prepared samples. 10](#_Toc183897294)

[Fig. S4. EPR spectra of e^-^ on C_11_H_10_FeO_2_^-^ 10](#_Toc183897295)

[Fig. S5 Steady-state PL spectra of as-prepared samples. 11](#_Toc183897296)

[Fig. S6 The UV-vis DRS spectra (a) and energy gap (b) of CSB and CSB-Fc NCs. 11](#_Toc183897297)

[Fig. S7 Density of states (DOS) and Partial density of states (PDOS) of as-prepared samples by DFT calculation 12](#_Toc183897298)

[Fig. S8. The photocurrent intensity of as-prepared samples. 12](#_Toc183897299)

[Fig. S9. EIS Nyquist plots of CSB and CSB-Fc NCs. 13](#_Toc183897300)

[Fig. S10 Optimized structures and sites tested for ligand exchange on the CSB surface. 13](#_Toc183897301)

[Fig. S11. Active sites tested for CSB-Fc surface. 14](#_Toc183897302)

[Fig. S12 The adsorption behavior and energy barrier of different molecules for photocatalytic reduction on the surface of CSB and CSB-Fc NCs via DFT calculation. 15](#_Toc183897303)

[Fig. S13 The adsorption behavior and energy barrier of different molecules for photocatalytic oxidation on the surface of CSB and CSB-Fc NCs via DFT calculation. 15](#_Toc183897304)

[Fig. S14 The HOMO and LUMO energy of Fc before and after CO_2_ adsorption. 15](#_Toc183897305)

[Fig. S15 Recycle test of performance over CSB-Fc. 16](#_Toc183897306)

[Fig. S16 The XRD diffraction of CSB-Fc before or after activity test. 16](#_Toc183897307)

[Fig. S17. In situ ATR-FTIR spectra of CSB and CSB-Fc samples upon passing CO_2_ + PHCH_2_OH atmosphere with adsorption process as time. 17](#_Toc183897308)

[Fig. S18. The standard curve of benzyl alcohol concentration. 17](#_Toc183897309)

[Fig. S19. The standard curve of benzaldehyde concentration. 17](#_Toc183897310)

[Fig. S20. The standard curve of benzoic acid concentration. 18](#_Toc183897311)

[Fig. S21. The designed reaction system for in situ Raman signal recording. 18](#_Toc183897312)

[Table S1. Fitting the average lifetime of time-resolved fluorescence spectra of as-prepared samples. 19](#_Toc183897313)

[Table S2. The detection concentration of respective products in the photocatalytic oxidation half-reaction 19](#_Toc183897314)

[Table S3. The detection yield of respective products in the photocatalytic oxidation half-reaction 19](#_Toc183897315)

[Table S4 AQE of CO_2_ photo-reduction with CSB-Fc 20](#_Toc183897316)

[Table S5. Summary of lately developed photocatalysts and their performance for the selective oxidation of benzyl alcohol and CO_2_ reduction. 21](#_Toc183897317)

[Table S6. DFT calculated reaction energy changes (ΔG) o 21](#_Toc183897402)

[References 22](#_Toc183897424)

Materials and Methods

Material Preparation: Oleic acid (OA, 90%) was purchased from Aldrich, antimony (III) tribromide (SbBr_3_, 99%, Sigma), 1-octadecene (ODE, tech, 90%, Aldrich), Oleylamine (OAm, tech, 80-90%, Aldrich), Octanoic acid (OnA, tech, 98%, Aladdin), and cesium carbonate (Cs_2_CO_3_, 99.99%, Aladdin). All of the chemical reagents of analytical grade were used as received without further purification.

Characterizations

The crystal phases of the samples were examined by X-ray diffraction (XRD) with Cu Kα radiation (model D/max RA, Rigaku Co., Japan). X-ray photoelectron spectroscopy (XPS) with Al Kα X-rays (XPS, Thermo ESCALAB 250, U.S.A.) was utilized to investigate the surface properties. The optical properties of the samples were investigated by UV-vis diffuse-reflectance spectrometry (UV-vis DRS), which was obtained for the dry-pressed disk samples, using a scanning UV-vis spectrophotometer (UV-vis DRS, UV-2450, Shimadzu, Japan) with an integrating sphere assembly and 100% BaSO_4_ as the reflectance sample. Photoluminescence (PL) studies (F-70007, HITACHI, Japan) were conducted to investigate the optical properties of the samples. Electron paramagnetic resonance (EPR) measurements were carried out on a Broker ESP 500 spectrometer at room temperature. Electron spin resonance (EPR) of spin-trapped by 2,2,6,6-Tetramethyl-1-piperidinyloxy (TEMPO) was recorded on a JES FA200 spectrometer at room temperature. Samples for ESR measurement were prepared by mixing the samples with a certain amount of TEMPO solution tank (aqueous dispersion for TEMPO-e^-^) and irradiated with visible light. Scanning electron microscopy (SEM, model JSM-6490, Japan) and transmission electron microscopy (TEM, JEM-2010, Japan) were used to characterize the morphology and structure.

DFT calculations

The molecular orbitals of CO_2_. Geometry optimization and single-point energy tasks were performed using the ORCA package (Version 5.0.4) and these DFT calculations were carried out with B3LYP exchange-correlation function and the def2-TZVP basis set. The molecular orbitals of CO_2_ molecules were visualized by Multiwfn software (Version 3.8). The Isosurface maps of Fukui function were rendered by VMD (Version 1.9.4) program.

Spin-polarized DFT-D2 computations were carried out using the Vienna Ab-initio Simulation Package (VASP). The exchange correlational functional was described with the generalized gradient approximation (GGA) method in the version of the Perdew-Burke-Ernzerhof (PBE) functional. All geometries were fully optimized with a plane wave energy cutoff of 400 eV and the electronic forces and energy were converged within 0.03 eV/ Å and 1×10^-5^ eV, respectively. The Gaussian smearing with a value of smearing parameter σ of 0.2 eV was applied. The Brillouin zone was sampled with 2 × 2 × 2 Monkhorst Pack k-point meshes. A pristine CSB (022) slab surface. The thickness of the vacuum layers was set to 15 Å. The adsorption energy (Eads) for reactants and molecules was calculated with the following equation:

| E_ads_ = E_tot_ - (E_mol_ + E_cata._) | (1) |
| --- | --- |

where E_tot_, E_mol_, and E_cata._ were the total energies of the adsorption structures, the isolated molecules, and the CSB (CSB) and CSB-Fc (CSB-Fc) NCs structures, respectively.

The Gibbs free energy of each reaction intermediate at 298 K was calculated using standard adsorbate and ideal-gas methods [1] :

| G = E_DFT_ + ZPE +$\int_{0}^{298} C_{P} dt-TS$ | (2) |
| --- | --- |

where $E_{DFT}$ is the electronic energy derived directly from the DFT calculations, ZPE is the zero-point energy, $C_{p}$ is the constant volume heat capacity and S denotes entropy of the species. Table S4 in the Supporting Information summarizes all the thermodynamic corrections.

Evaluation of photo-redox activity

The CO_2_ photoreduction performances were carried out in a reactor under a 300 W Xe lamp with a 400 nm cut-off filter (PLS-SEX300C, Beijing Perfect-light Technology) light source, and the gas phase products were analyzed with a Labsolar 6A closed circulation system (Beijing Perfect light Technology Co., Ltd., China). Typically, the photocatalyst (10 mg) was suspended in acetonitrile (1 mL) containing benzyl alcohol (BA) (0.01 mmol) in a reaction chamber. Subsequently, the pure CO_2_ was injected into the reactor at a constant pressure of about 1 atm after the reaction system was evacuated several times. The reaction temperature was kept at 298 K using a circulation cooling system (DC-0506, Shanghai Sunny Hengping Scientific Instrument Co., China). A GC 2002 gas chromatograph (Shanghai Kechuang Chromatography Instrument Co., Ltd., China) was used to analyze the products qualitatively. The CO selectivity was calculated according to the required electrons for CO_2_ reduction as follows:

CO selectivity (%) = [2φ(CO)]/[2φ(CO) + 8φ(CH_4_) + 2φ(H_2_)] × 100%.

where φ(CO), φ(CH_4_), and φ(H_2_) represent the yield rate of the products, respectively.

In addition, 0.1 mL of the above solution was taken, centrifuged and filtered through a 0.22-mm nylon syringe filter. The concentration of BA was measured using high-performance liquid chromatography (Shimadzu LC-20A, Japan, TC-C18 reverse phase column). We have done the standard curves of each component (such as benzyl alcohol, benzoic acid and benzaldehyde), respectively, as shown in Figure S16-18. The conversion percentage of BA and the selectivity for benzaldehyde (BD) are defined as:

Conversion (%) = [(C_BD_ + C_BO_ + C_BA_)/C_0_] × 100%;

Selectivity (%) = [C_BD_/ (C_BD_ + C_BO_ + C_BA_)] × 100%,

where C_0_ is the initial concentration of BA. C_BO_ C_BA_ and C_BD_ are the concentrations of the detected benzoic acid, benzyl alcohol and benzaldehyde, respectively.

The ratio of electrons and holes consumed in redox reactions was calculated by the following equation: e^–^/h^+^ = [2*φ(H_2_) + 2*φ(CO)+8*φ(CH_4_)]/ [ φ(BO) + φ(BD)].

In situ Raman tests

A total of 10 mg of catalyst and reactant solution were uniformly dispersed into a 2 cm × 2 cm × 1 cm quartz vessel. In-situ liquid Raman experiments were conducted using a laser Raman spectrometer (Soleil, HORIBA) with a 633 nm laser to collect data in the range of 50 to 400 cm^-1^ (Figure S21).


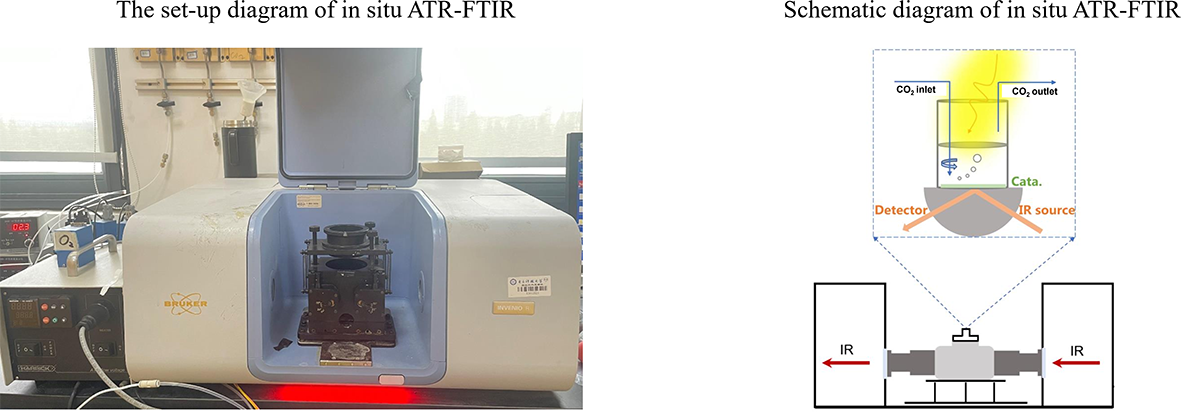


Scheme S1. The designed reaction system for in situ ATR-FTIR signal recording.


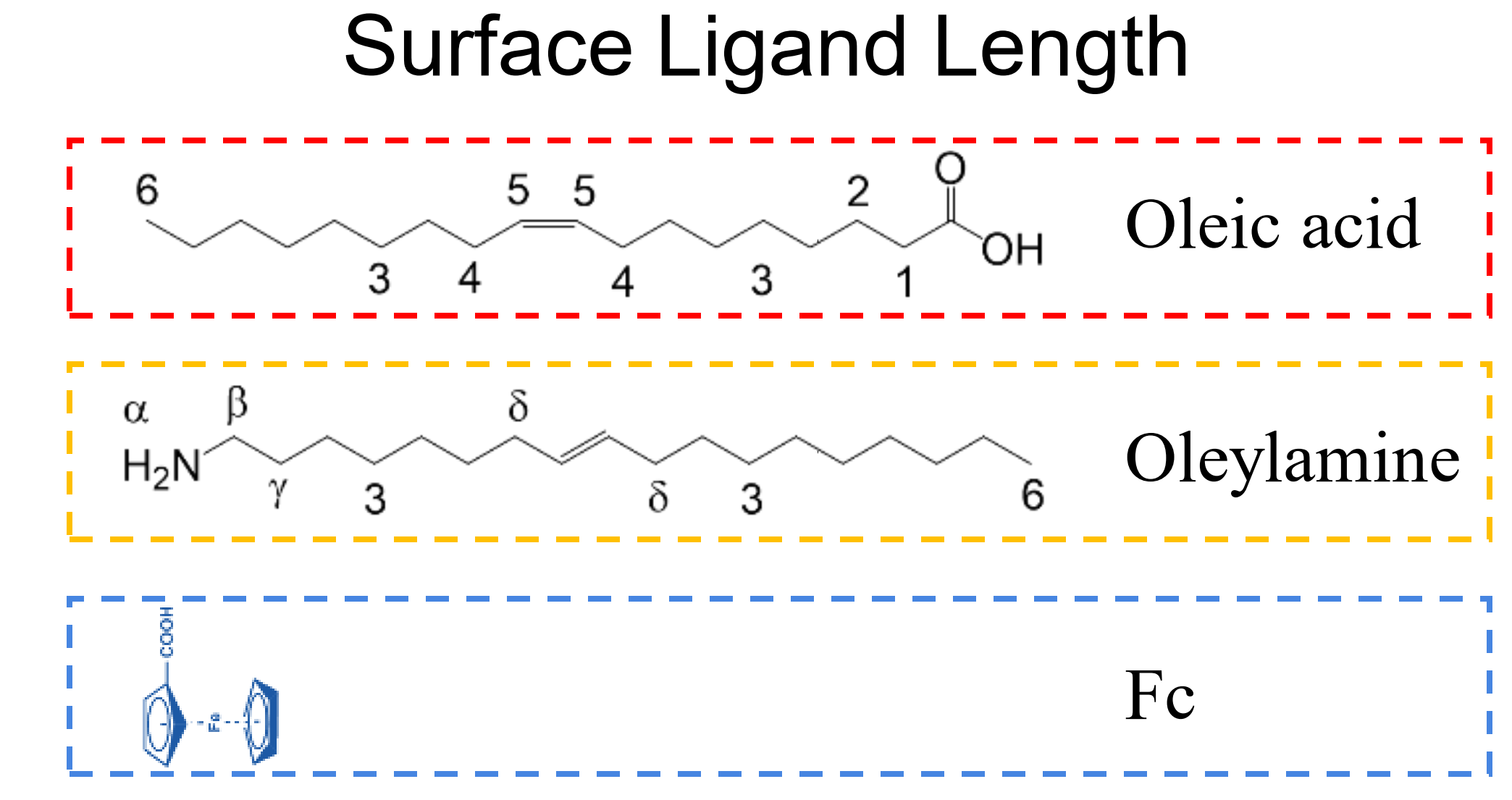


Fig. S1. The surface ligand length.


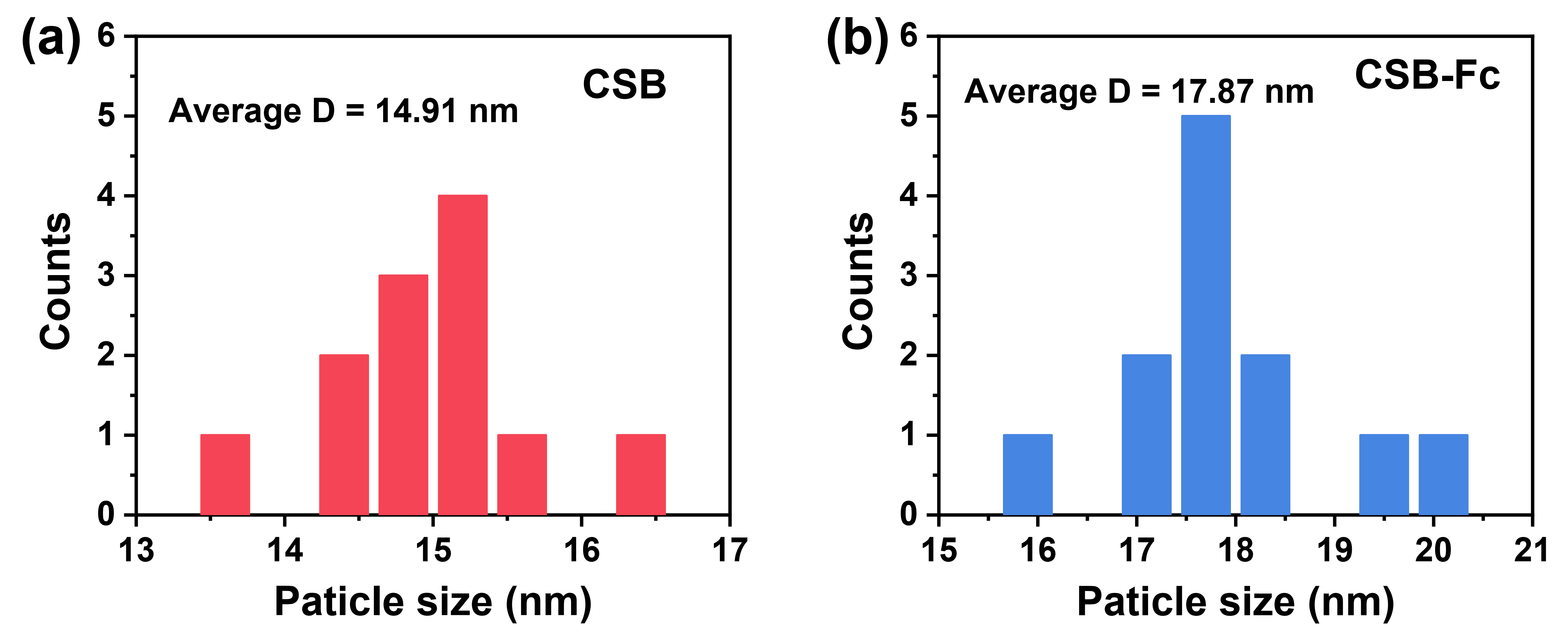


Fig. S2. Analysis of the size distribution over CSB and CSB-Fc NCs


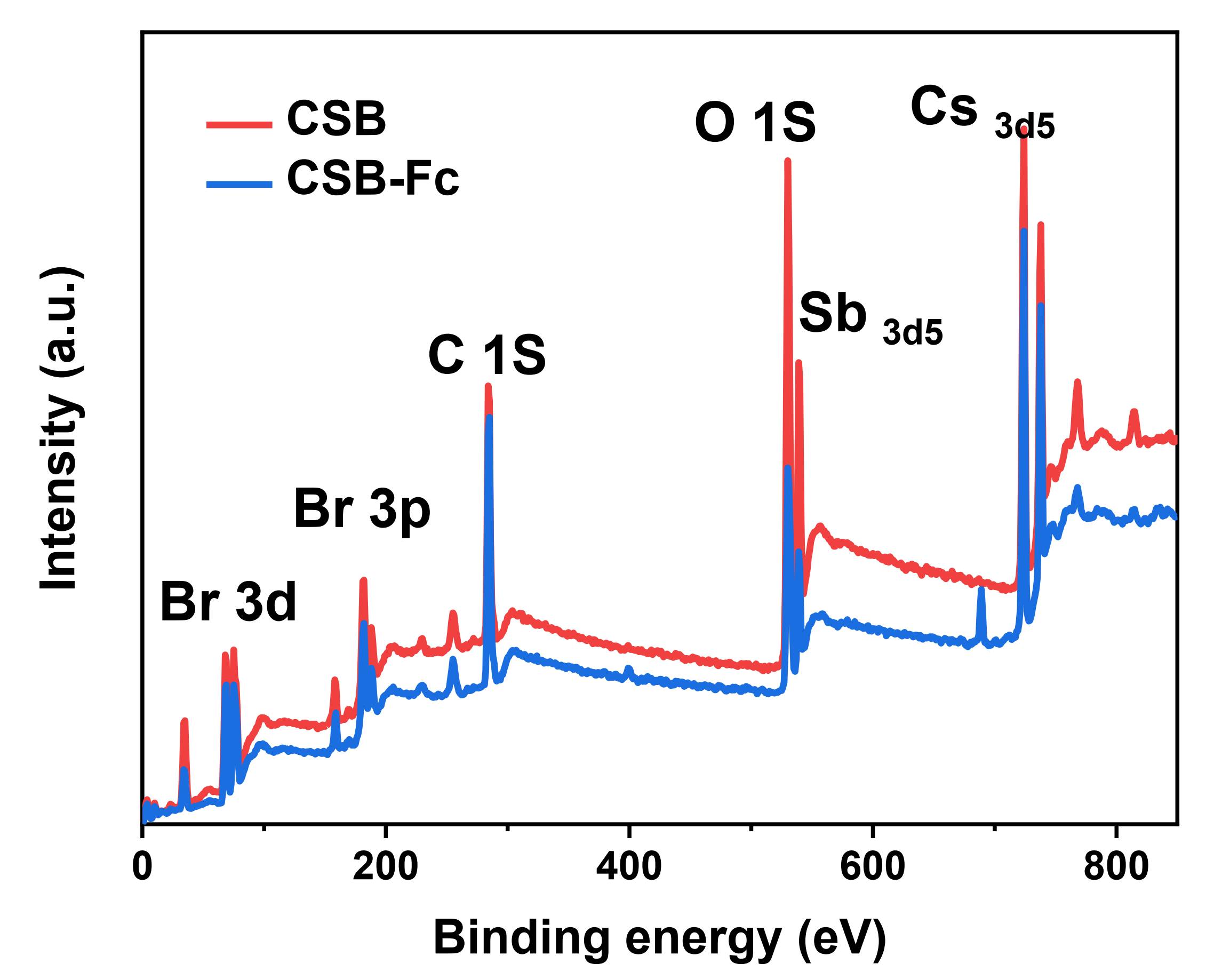


Fig. S3. The survey spectra of as-prepared samples.


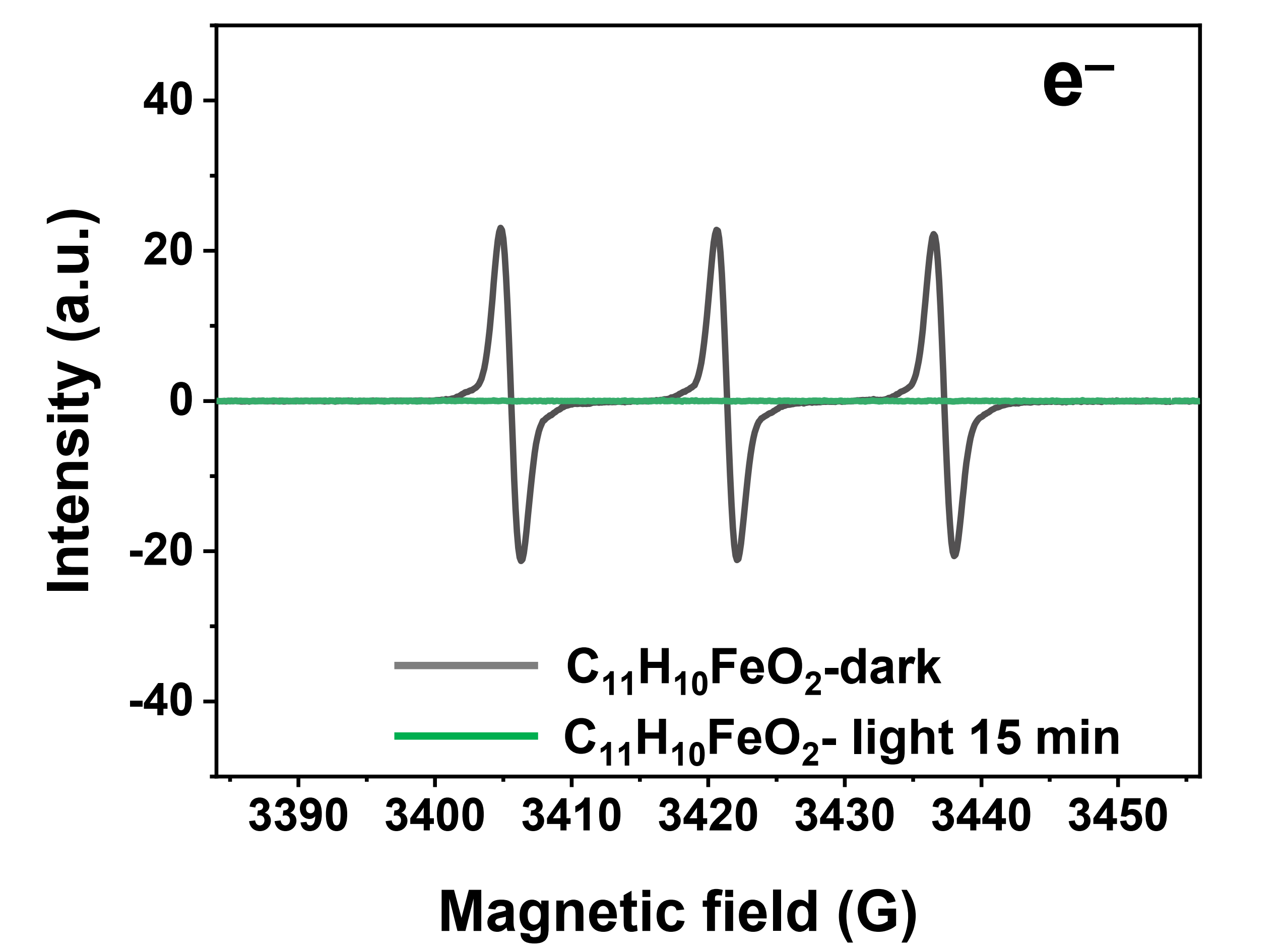


Fig. S4. EPR spectra of e^-^ on C_11_H_10_FeO_2_^-^


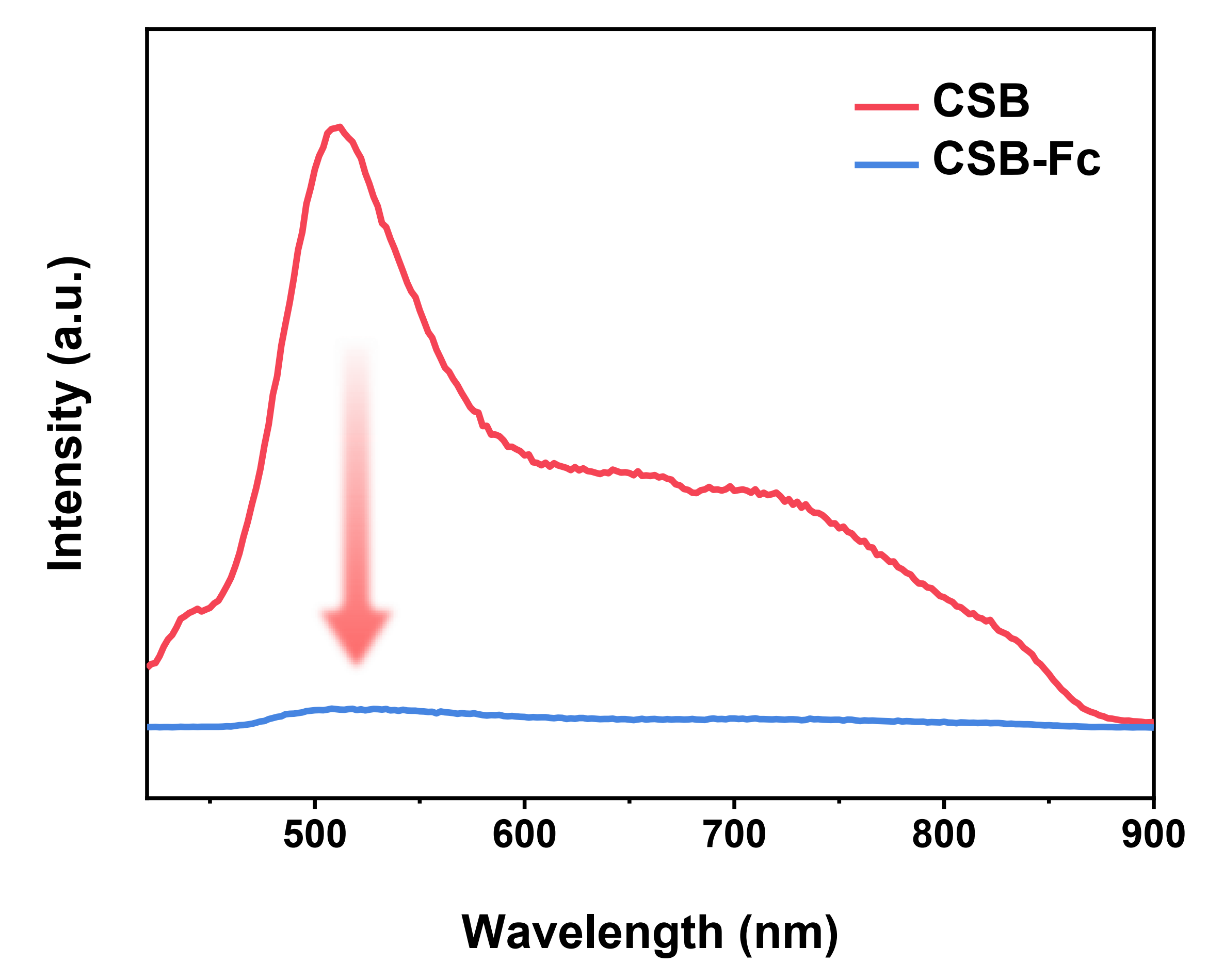


Fig. S5 Steady-state PL spectra of as-prepared samples.


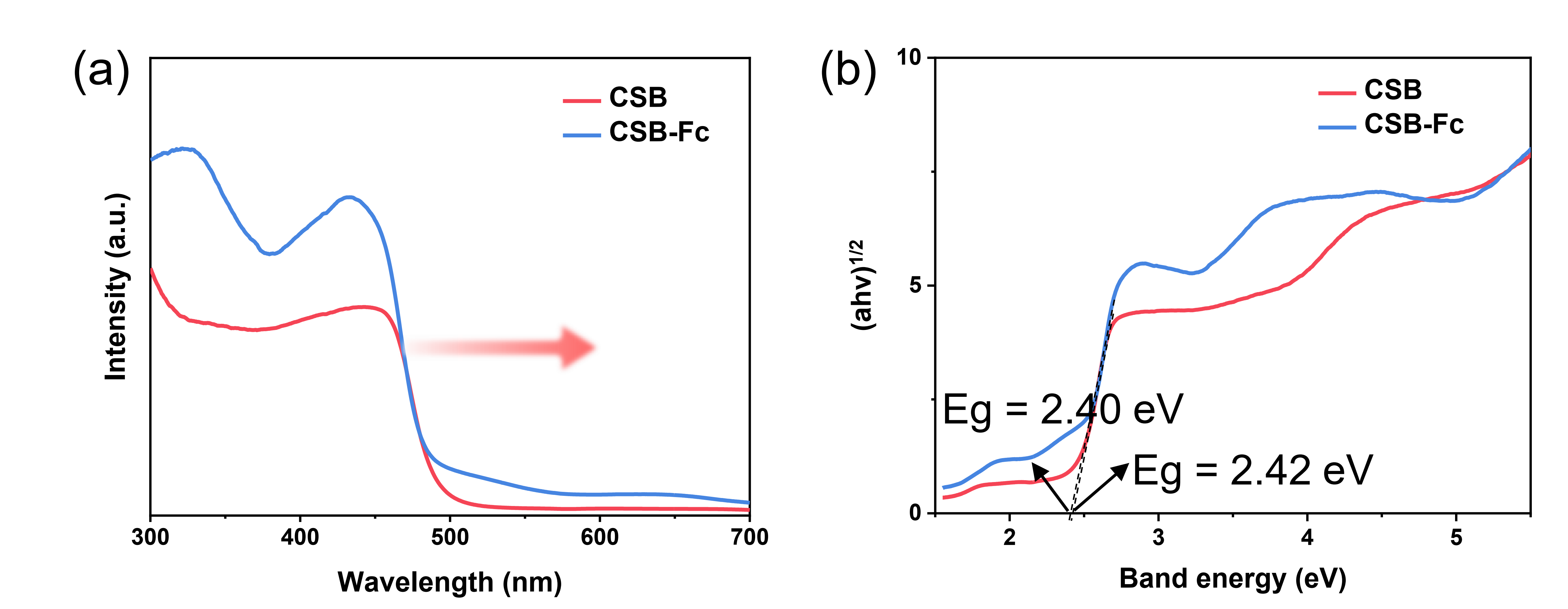


Fig. S6 The UV-vis DRS spectra (a) and energy gap (b) of CSB and CSB-Fc NCs.


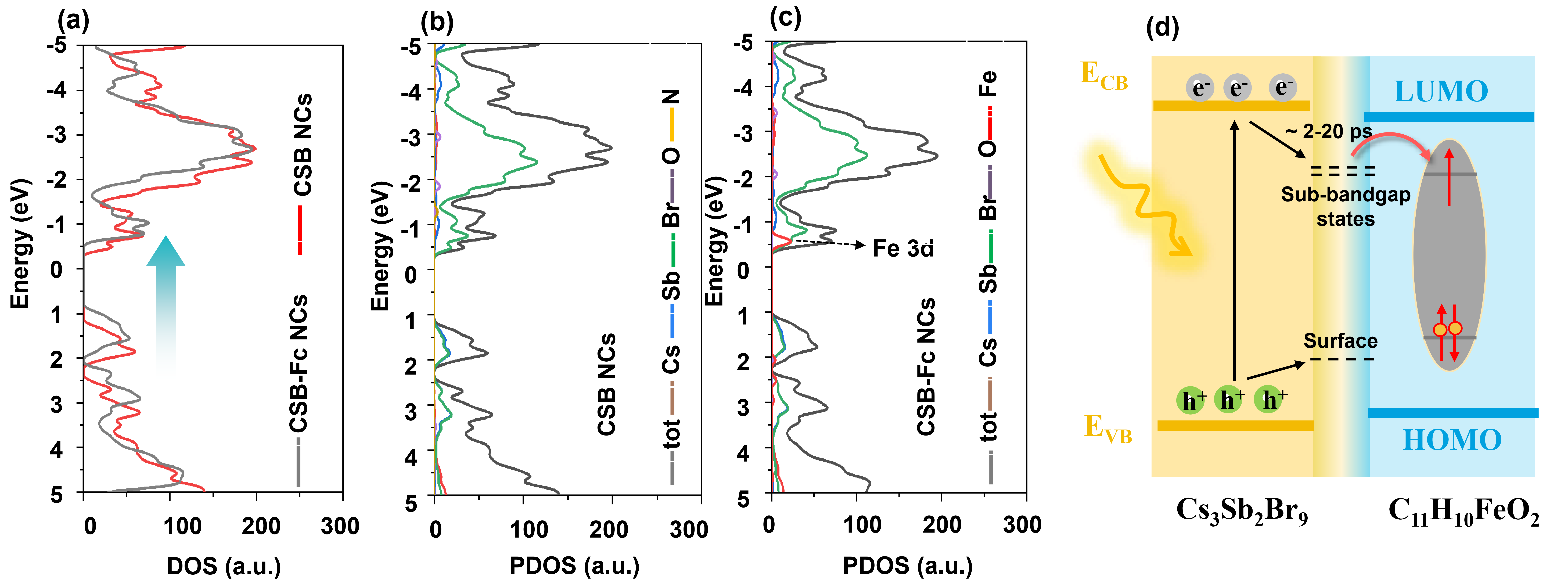


Fig. S7 Density of states (DOS) and Partial density of states (PDOS) of as-prepared samples by DFT calculation


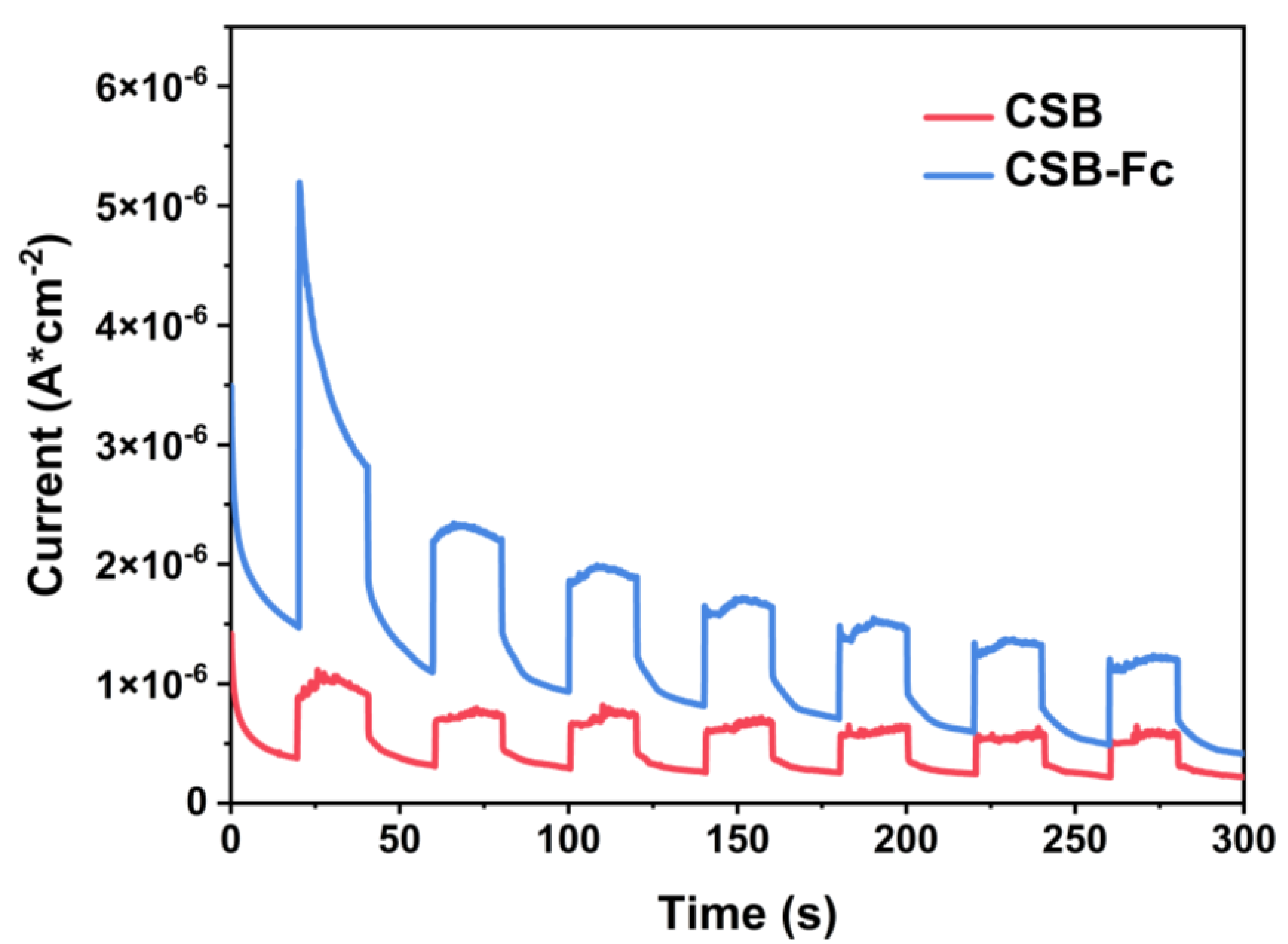


Fig. S8. The photocurrent intensity of as-prepared samples.


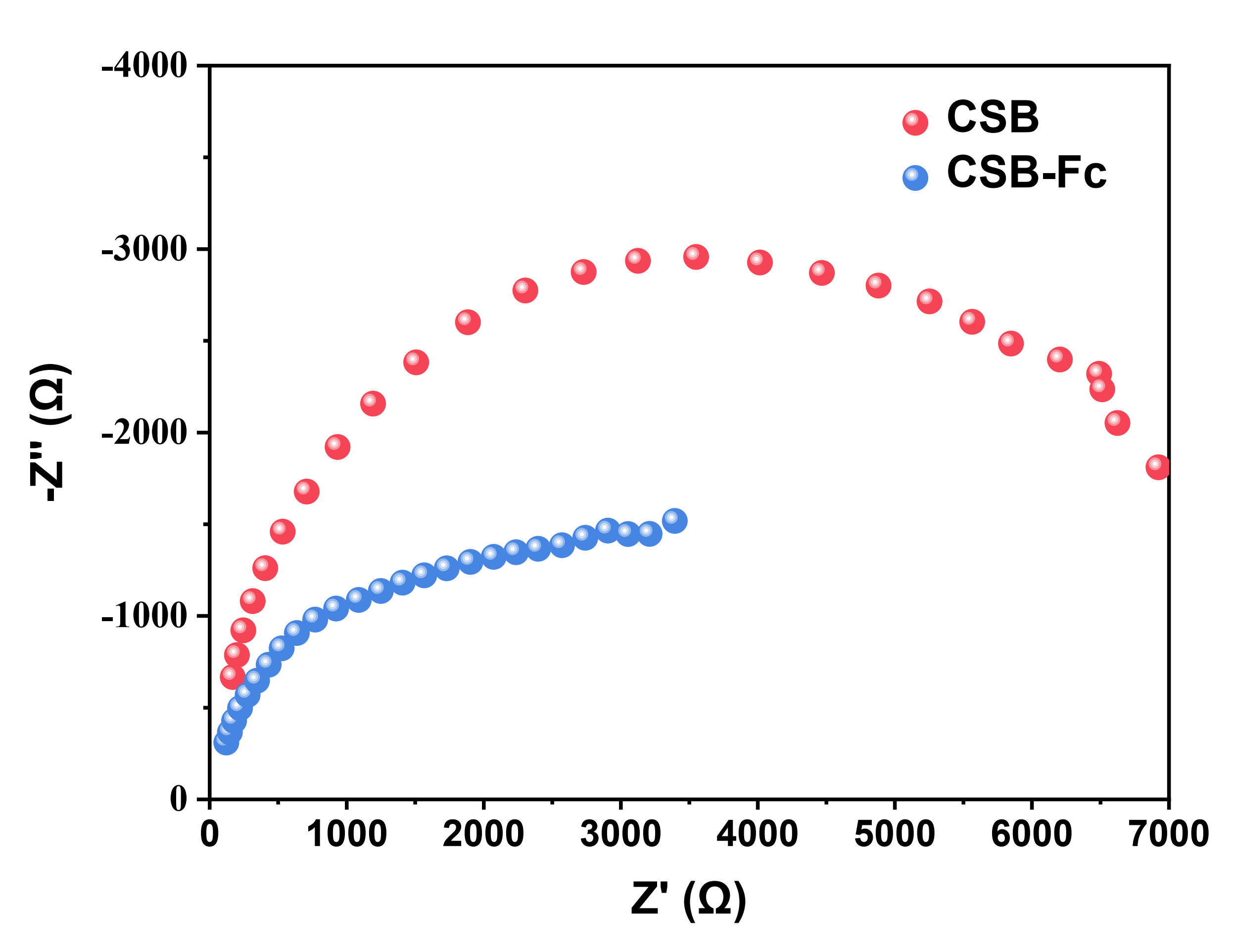


Fig. S9. EIS Nyquist plots of CSB and CSB-Fc NCs.

Fig. S10 Optimized structures and sites tested for ligand exchange on the CSB surface.

Fig. S11. Active sites tested for CSB-Fc surface.

Fig. S12 The adsorption behavior and energy barrier of different molecules for photocatalytic reduction on the surface of CSB and CSB-Fc NCs via DFT calculation.

Fig. S13 The adsorption behavior and energy barrier of different molecules for photocatalytic oxidation on the surface of CSB and CSB-Fc NCs via DFT calculation.


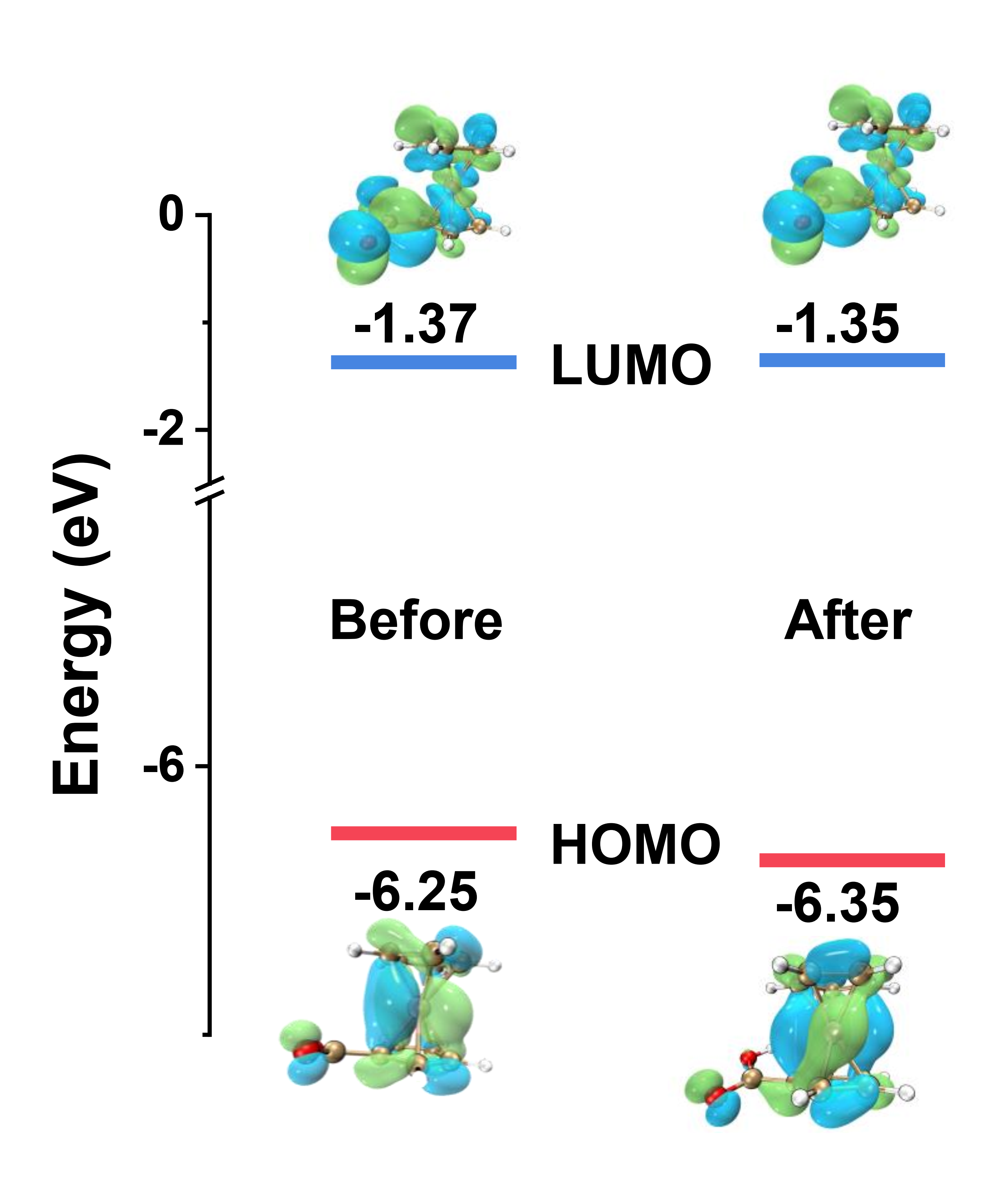


Fig. S14 The HOMO and LUMO energy of Fc before and after CO_2_ adsorption.


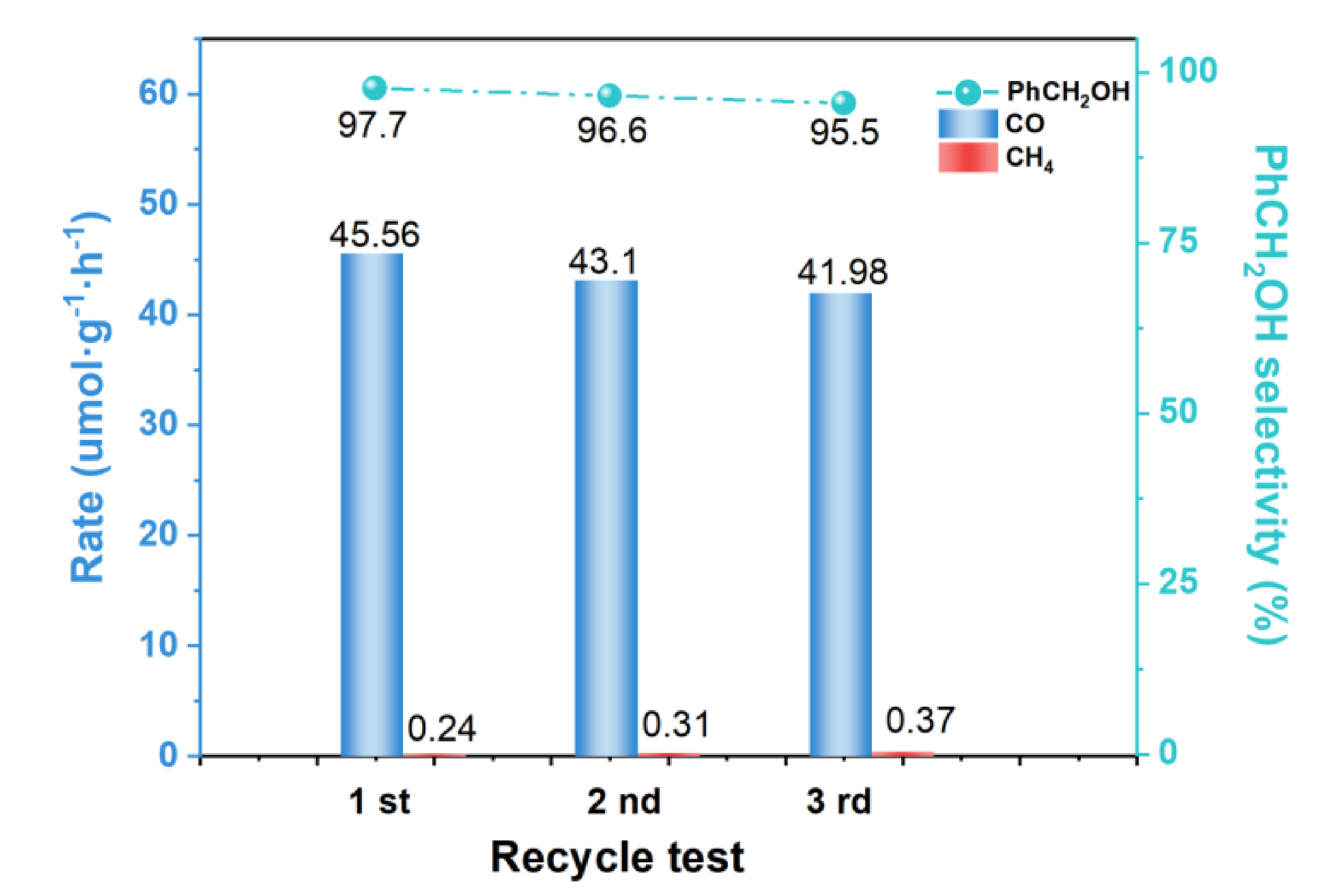


Fig. S15 Recycle test of performance over CSB-Fc.


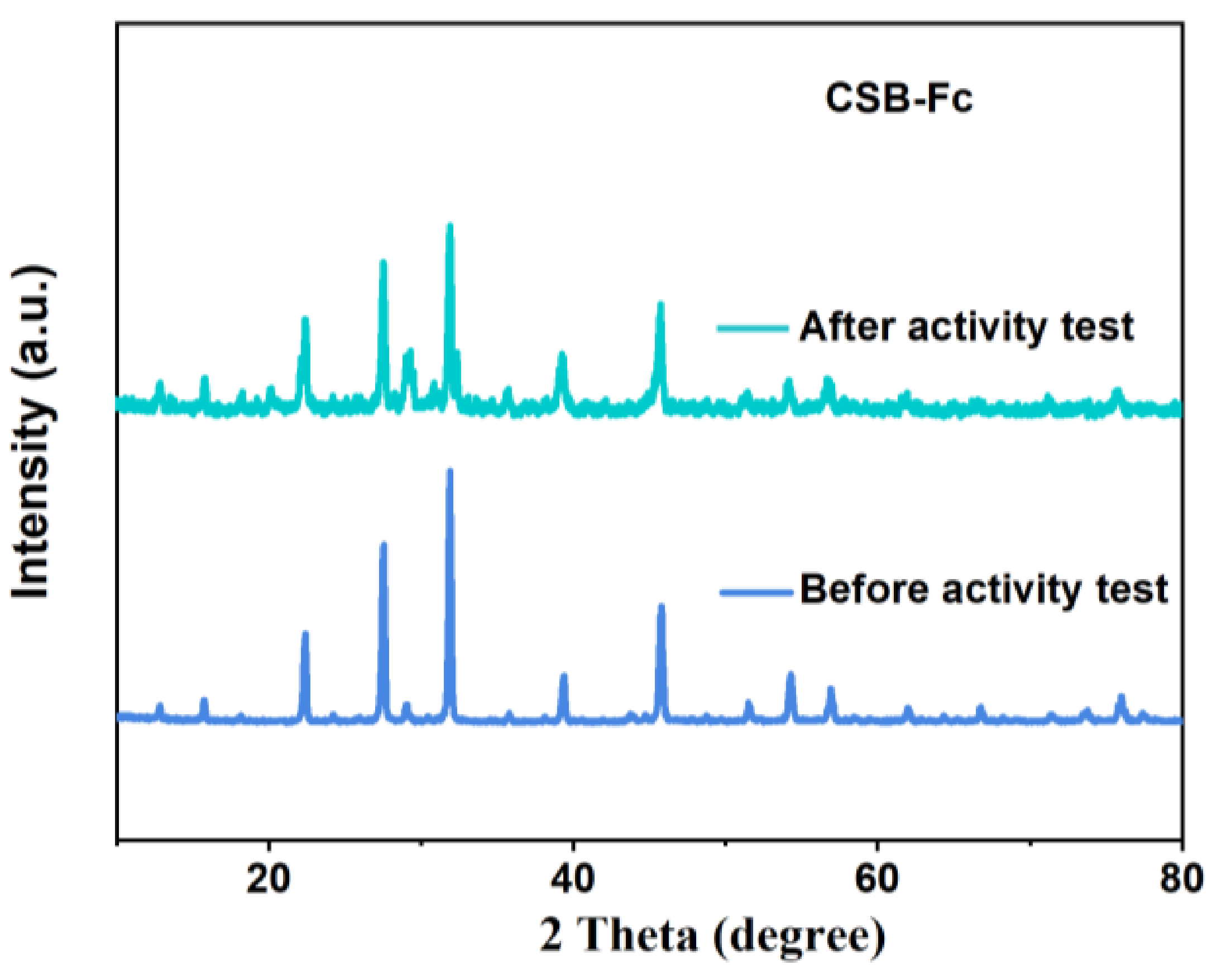


Fig. S16 The XRD diffraction of CSB-Fc before and after activity test.


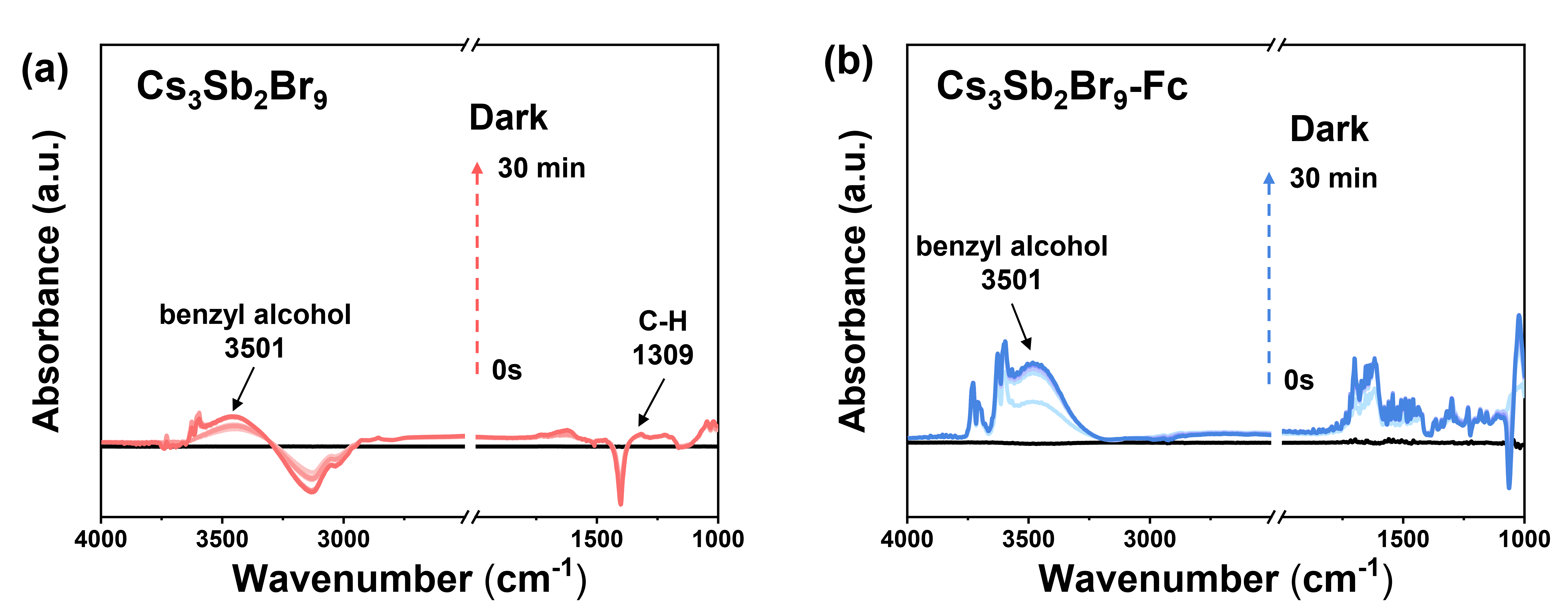


Fig. S17. In situ ATR-FTIR spectra of CSB and CSB-Fc samples upon passing CO_2_ + PHCH_2_OH atmosphere with adsorption process as time.


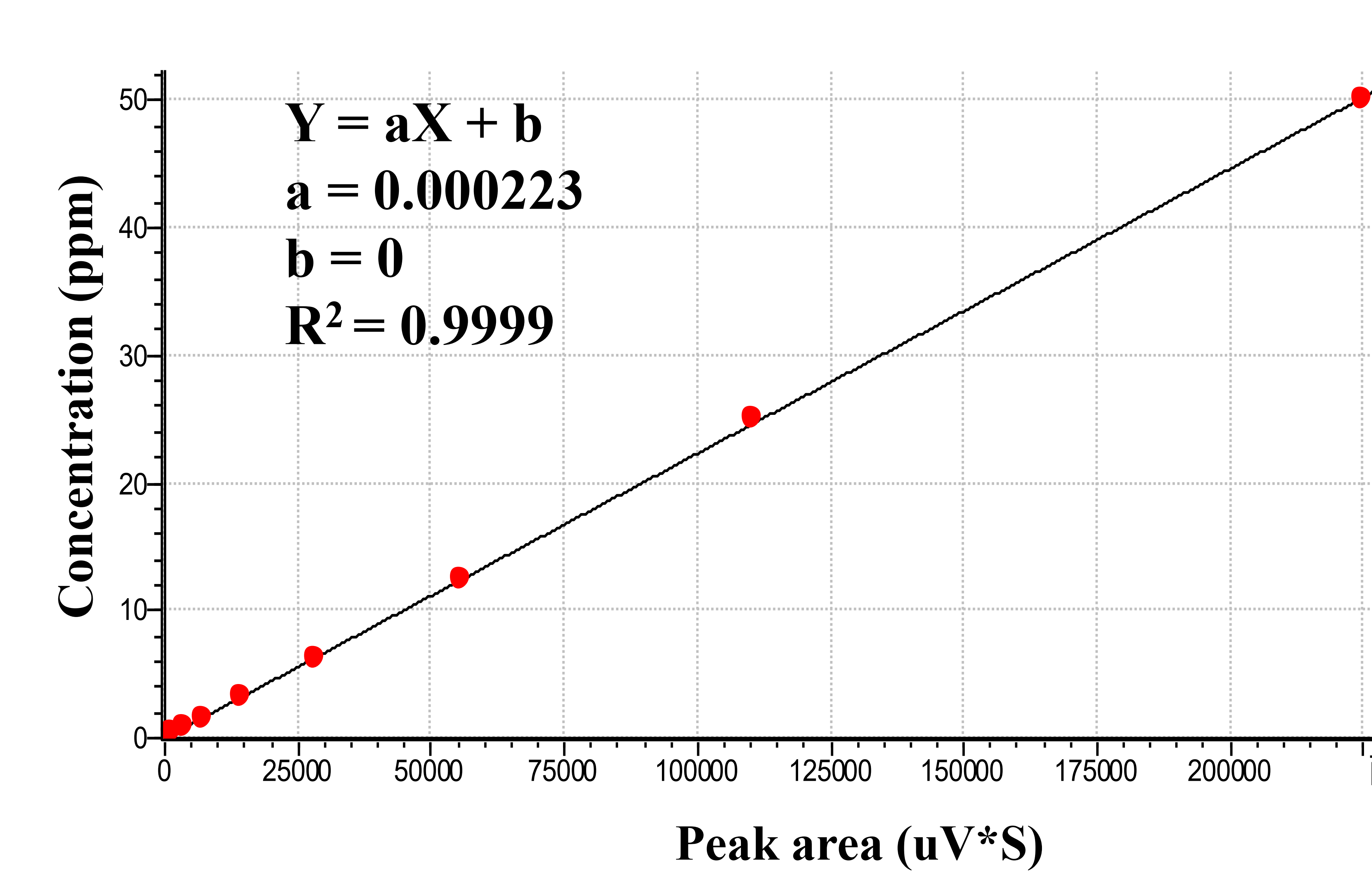


Fig. S18. The standard curve of benzyl alcohol concentration.


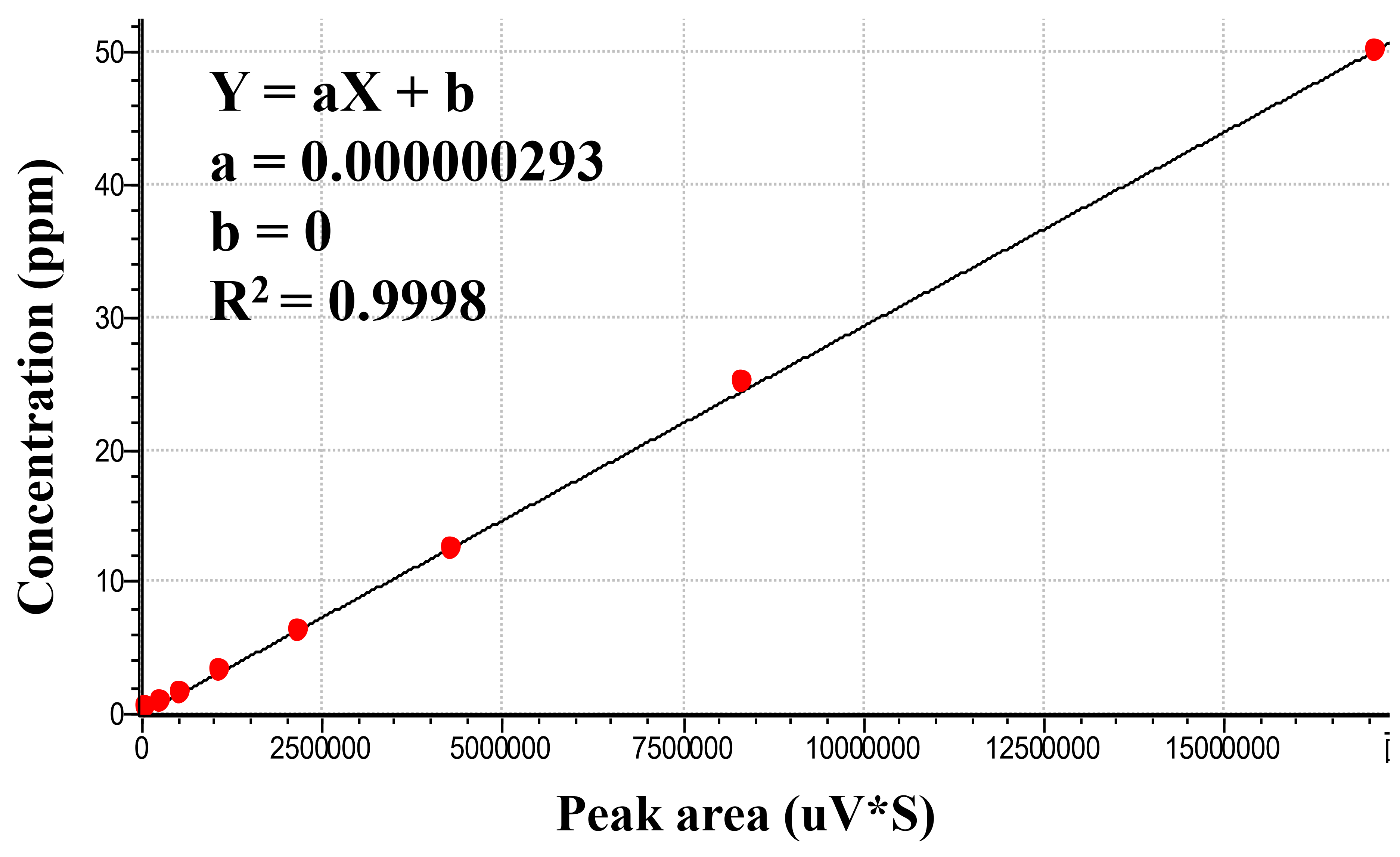


Fig. S19. The standard curve of benzaldehyde concentration.


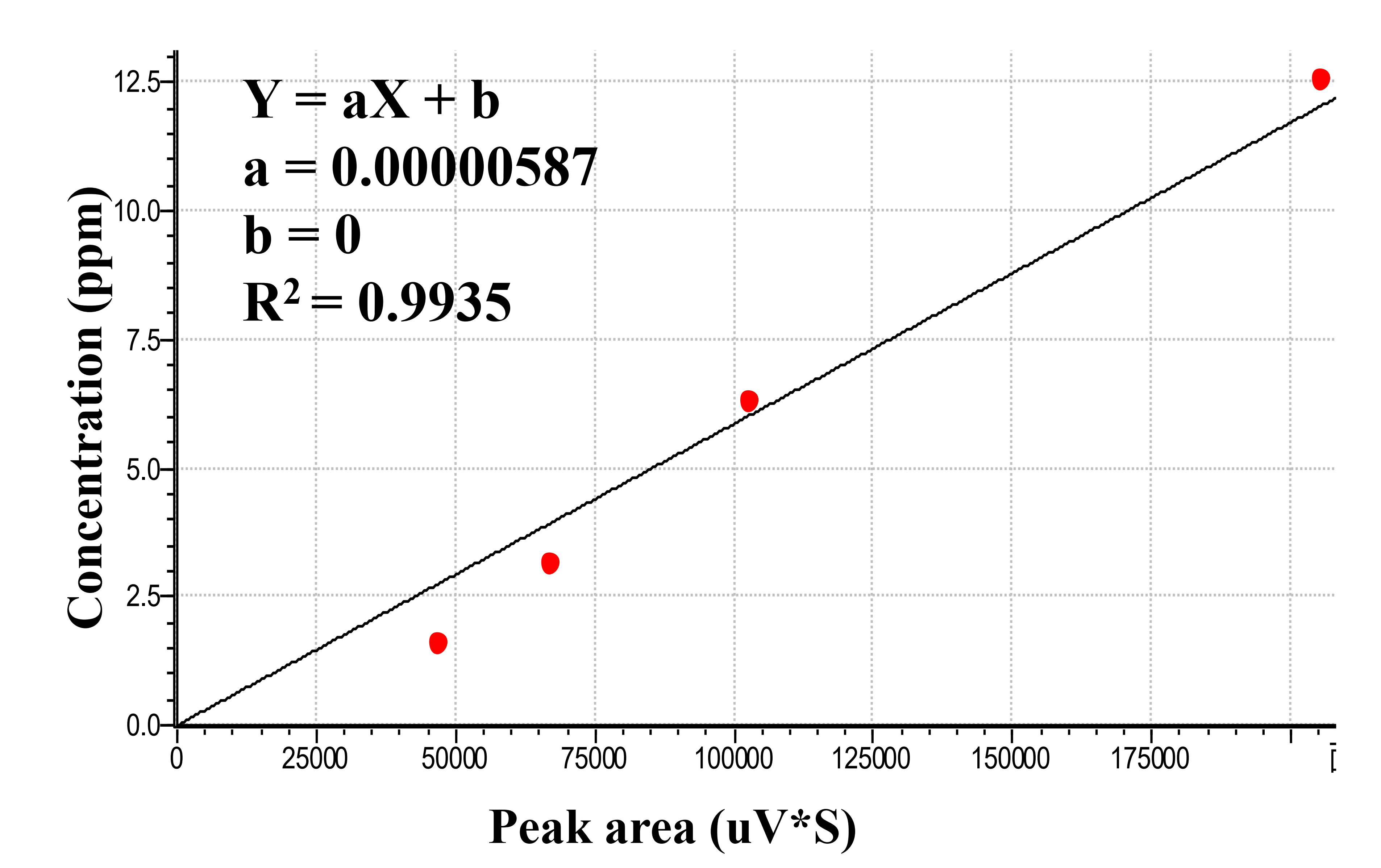


Fig. S20. The standard curve of benzoic acid concentration.


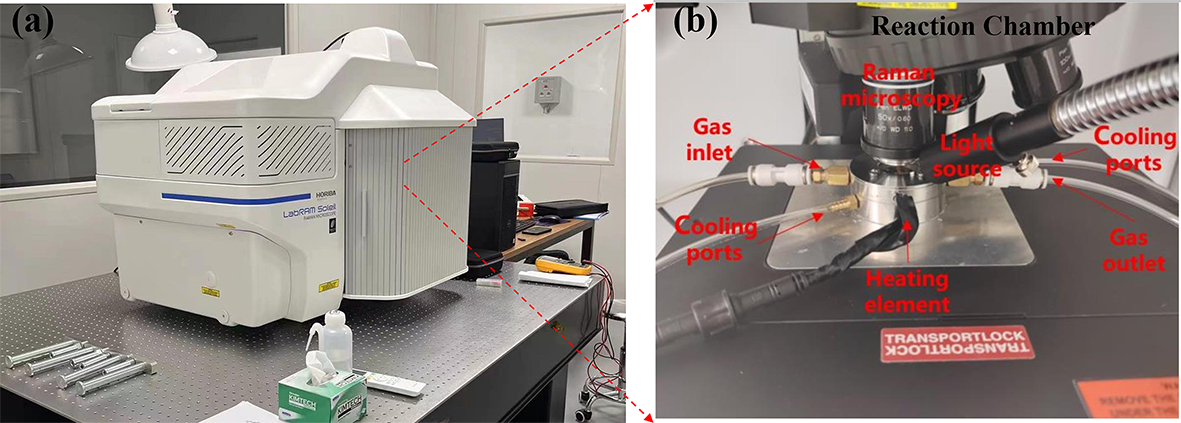


Fig. S21. The designed reaction system for in situ Raman signal recording.

Table S1. Fitting the average lifetime of time-resolved fluorescence spectra of as-prepared samples.

| Entry | Catalyst | τ_1_ | τ_2_ | τ_avg._ |
| --- | --- | --- | --- | --- |
| 1 | CSB NCs | 5.91 | 0.46 | 1.40 |
| 2 | CSB-Fc NCs | 4.81 | 0.43 | 0.46 |

Table S2. The detection concentration of respective products in the photocatalytic oxidation half-reaction

| Entry | Catalyst | C_0_ (mg/ml) | Time (h) | C_BO_  (mg/ml) | C_BD_  (mg/ml) | BO Sel.  (%) | BD  Sel. (%) | BA  Con. (%) |
| --- | --- | --- | --- | --- | --- | --- | --- | --- |
| 1 | CSB | 12.023 | 4 | 0.13 | 0.743 | 14.8 | 85.1 | 7.1 |
| 2 | CSB-Fc | 12.97 | 4 | 0.10 | 5.504 | 2.3 | 97.69 | 43.5 |

where C_0_ is the initial concentration of BA. C_BO_ C_BA_ and C_BD_ are the concentrations of the detected benzoic acid, benzyl alcohol and benzaldehyde, respectively.

Table S3. The detection yield of respective products in the photocatalytic oxidation half-reaction

| Entry | Catalyst | Time (h) | CO  (µmol·g^-1^·h^-1^) | CH_4_  (µmol·g^-1^·h^-1^) | H_2_  (µmol·g^-1^·h^-1^) | CO sel.  (%) |
| --- | --- | --- | --- | --- | --- | --- |
| 1 | CSB | 4 | 45.56 | 0.24 | 0 | 78.56 |
| 2 | CSB-Fc | 4 | 8.745 | 0.59 | 0 | 97.82 |

Table S4 AQE of CO_2_ photo-reduction with CSB-Fc

| Wavelength (nm) | 450 | 500 | 600 |
| --- | --- | --- | --- |
| CO production (umol) | 2.645 | 0.7228 | 0.8667 |
| Light intensity (mW/cm^2^) | 91.2 | 93.92 | 95.28 |
| Irradiation time (h) | 2 | 2 | 2 |
| Irradiation area (cm^2^) | 4 | 4 | 4 |
| AQE (%) | 0.0535 | 0.0126 | 0.0128 |

To obtain a more uniform irradiation spot, we used High Uniformity Integrated Optical Fiber Light source, PLS-SXE300UV, Beijing Perfect light Technology Co., Ltd.

λ = 450 nm:

The number of incident photons:

$N=\frac{E\lambda}{hc}$ = $\frac{91.2\times4 \times{10}^{-3} \times2 \times3600 \times400 \times{10}^{-9}}{6.626 \times{10}^{-34} \times3 \times{10}^{8}}=5.946\times{10}^{21}$

AQE ：

$$AQE =\frac{2 \times the number of CO production}{N} \times100 \%$$

$$= \frac{2 \times6.02 \times{10}^{23}\times2.645 \times{10}^{-9}}{5.946\times{10}^{21}}= 0.0535 \%$$

Table S5. Summary of lately developed photocatalysts and their performance for the selective oxidation of benzyl alcohol and CO_2_ reduction.

| Entry | Catalyst | CO Selectivity (%) | CO Rate  (µmol·g-1·h-1) | PhCH2OH Conversion (%) | PhCHO Selectivity (%) | e-/h+ | Ref. |
| --- | --- | --- | --- | --- | --- | --- | --- |
| 1 | HESO NFs | 11.2 | 8.03 | / | / | / | [2] |
| 2 | Ag/Bi_4_Ti_3_O_12_ | 53.2 | 34.15 | / | / | / | [3] |
| 3 | TiO_2_/CsPbBr_3_ | 95 | 9.02 | / | / | / | [4] |
| 4 | S-vacancies SnS_2_ | 57 | 25.71 | / | / | / | [5] |
| 5 | Ag1@PCN | 94 | 0.32 | / | / |  | [6] |
| 6 | CdS/Mo_2_C | 98.3 | 29.2 | / |  | / | [7] |
| 7 | Cu-TiO_2_ | 95.9 | 15.27 | / |  | / | [8] |
| 8 | Ni-SA-x/ZrO_2_ | 92.5 | / | / | 11.8 | / | [9] |
| 9 | Au_43_/Al_2_O_3_ | / | / | 32 | 98.3 |  | [10] |
| 10 | Holey HEO (0.9Ni) | / | / | 29 | 98 |  | [11] |
| 11 | CdS/TNS | ＞99.9 | 5.1 | 27.9 | 9.6 | / | [12] |
| 12 | CSB-Fc NCs | 97.7 | 45.56 | 41.5 | 97.7 | 0.94 | This work |

Table S6. DFT calculated reaction energy changes (ΔG) of conversion of CO_2_ photoreduction pathways and PhCH_2_OH oxidation over as-prepared samples, zero-point energy correction, enthalpic temperature correction, and entropy correction are included in total free energy.

| Catalyst | *CO_2_ | *COOH | *CO | CO | *PhCH_2_OH | *PhCHO | PHCHO |
| --- | --- | --- | --- | --- | --- | --- | --- |
| CSB | 0.06 eV | 2.428 eV | -1.80eV | -0.12 eV | -0.01 eV | 0.32 eV | 1.806 eV |
| CSB-Fc | 0.018eV | 2.39 eV | -1.83eV | -0.14 eV | -0.209 eV | 0.36 eV | -0.653 eV |

References

[1] a) J. K. Nørskov, J. Rossmeisl, A. Logadottir, L. Lindqvist, J. R. Kitchin, T. Bligaard, H. Jónsson, *The Journal of Physical Chemistry B* 2004, *108*, 17886-17892; b) C. J. Cramer, West Sussex, England; New York: J. Wiley, [2002] ©2002, 2002.

[2] L. Zhang, S. Xia, X. Zhang, Y. Yao, Y. Zhang, S. Chen, Y. Chen, J. Yan, *ACS Nano* 2024, *18*, 5322-5334.

[3] L. Liu, J. Hu, Z. Ma, Z. Zhu, B. He, F. Chen, Y. Lu, R. Xu, Y. Zhang, T. Ma, M. Sui, H. Huang, *Nat. Commun.* 2024, *15*, 305.

[4] F. Xu, K. Meng, B. Cheng, S. Wang, J. Xu, J. Yu, *Nat. Commun.* 2020, *11*, 4613.

[5] S. Yin, X. Zhao, E. Jiang, Y. Yan, P. Zhou, P. Huo, *Energy Environ. Sci.* 2022, *15*, 1556-1562.

[6] S. Hu, P. Qiao, X. Yi, Y. Lei, H. Hu, J. Ye, D. Wang, *Angew. Chem. Int. Ed.* 2023, e202304585.

[7] Y.-L. Men, Y. You, Y.-X. Pan, H. Gao, Y. Xia, D.-G. Cheng, J. Song, D.-X. Cui, N. Wu, Y. Li, S. Xin, J. B. Goodenough, *J. Am. Chem. Soc.* 2018, *140*, 13071-13077.

[8] K. Zhu, Q. Zhu, M. Jiang, Y. Zhang, Z. Shao, Z. Geng, X. Wang, H. Zeng, X. Wu, W. Zhang, K. Huang, S. Feng, *Angew. Chem. Int. Ed.* 2022, *61*, 202207600.

[9] X. Xiong, C. Mao, Z. Yang, Q. Zhang, G. I. N. Waterhouse, L. Gu, T. Zhang, *Adv. Energy Mater.* 2020, *10*, 2002928.

[10] Y. Li, H. K. Kim, R. D. McGillicuddy, S.-L. Zheng, K. J. Anderton, G. J. Stec, J. Lee, D. Cui, J. A. Mason, *J. Am. Chem. Soc.* 2023, *145*, 9304-9312.

[11] D. Feng, Y. Dong, L. Zhang, X. Ge, W. Zhang, S. Dai, Z. A. J. A. C. Qiao, *Angew.Chem. Int. Ed* 2020, *132*, 19671-19677.

[12] M. Y. Qi, Q. Lin, Z. R. Tang, Y. J. Xu, *Appl. Catal., B* 2022, *202*.
